# On the Improvement of the Sterile Insect Technique by Entomopathogenic Fungi: Impact of Residual Fertility and Re-mating Behaviour

**DOI:** 10.1007/s11538-025-01529-8

**Published:** 2025-09-17

**Authors:** Yves Dumont

**Affiliations:** 1 CIRAD, Umr AMAP, Pôle de Protection des Plantes, CIRAD, Saint Pierre, F-97410 France; 2 AMAP, Univ Montpellier, CIRAD, CNRS, INRAe, IRD, Montpellier, France; 3 Department of Mathematics and Applied Mathematics, University of Pretoria, Pretoria, South Africa

**Keywords:** Sterile Insect Technique, Residual fertility, Entomopathogenic Fungi, Beauveria Bassiana, Re-mating, Refractory period, Elimination, Mathematical Modelling, Monotone dynamical systems, Threshold parameters, Continuous releases, Periodic releases, Medfly, Oriental fruit fly, Numerical simulations

## Abstract

This study investigates the use of the Sterile Insect Technique (SIT) combined with Entomopathogenic Fungi soil treatment (EPFS) to control two major pests: the Mediterranean fruit fly and the Oriental fruit fly. The SIT involves releasing sterile males to mate with wild females, but the challenge lies in female polyandry (re-mating) and residual fertility in sterile males. We develop a continuous release SIT model with single- and double-mated females, but with a novel approach to accounting the residual fertility parameter, $\varepsilon$. We also consider scenarios where the competitiveness of sterile males may decline between the first and the second mating. A key finding is that insect elimination, at least locally, with SIT can only occur when the product of the residual fertility parameter, $\varepsilon$, and the basic reproduction number of sterile mated females, $\mathcal {R}_S$, is less than 1. We also prove the existence of a sterile male release threshold, above which global elimination is possible. When $\varepsilon \mathcal {R}_S$ is greater than one, elimination is impossible regardless of the size of sterile male releases. We also extend our results to periodic releases. We illustrate our theoretical findings using numerical simulations, with parameters from the Mediterranean fruit fly (medfly), with and without ginger root oil (GRO) treatment, and the oriental fruit fly, with and without Methyl-Eugenol (ME) treatment. Both treatments are known to enhance sterile male competitiveness. We also show that combining SIT with EPFS can greatly improve SIT efficiency, and, in particular, reduce the constraint on residual fertility. We conclude that re-mating and residual fertility can have a significant impact on the effectiveness of SIT. However, this mainly depends on whether SIT is used in combination with EPFS or not, and also on the knowledge of the parameters of sterile-mated females which seem to have been superficially studied in many SIT programs so far.

## Introduction

The Sterile Insect Technique (SIT) is an autocidal genetic control yvesmethod used to manage, suppress, or eradicate agricultural pests and disease vectors across various countries (Krafsur 1998; Dyck et al. January 2021; Pérez-Staples et al. 2021). Among these pest, two are particularly threatening to orchards and crops: the Mediterranean fruit fly, *Ceratitis capitata*, already established in all continents (Giunti et al. 2023), and the Oriental fruit fly, *Bactrocera dorsalis*, that is considered one of the world’s most invasive species (Mutamiswa et al. 2021; Nugnes et al. 2024). Major SIT operational programs have been developed or are ongoing against the Mediterranean fruit fly (Dyck et al. January 2021; Pérez-Staples et al. 2021), and, to a lesser extent, the Oriental fruit fly (Sutantawong et al. 2004). In fact, the first investigations about SIT against *Ceratitis capitata* and *Bactrocera dorsalis* started in Hawaii in 1955 (Dyck et al. January 2021)[Chapter 1]. SIT requires the mass rearing of the target insect, followed by the sterilization of males only (or both males and females, depending on the species) either as pupae or adults using ionizing radiation. These sterilized insects are then released in large numbers into the field, where they mate with wild populations, leading to a gradual decline in the pest population over time (Knipling 1955; Dyck et al. January 2021; Pérez-Staples et al. 2021). While conceptually very simple, SIT can be difficult to deploy on the field as it requires a very good knowledge of the target insect and also to master all technical issues related to mass rearing and sterilization. That is why it is important to have feasibility programs. Now, in France, in Réunion island, a SIT research feasibility project (called AttracTIS) is ongoing to determine if SIT can be efficient against the oriental fruit fly (Moquet et al. 2024). Another SIT program, against the same pest, is also ongoing in Thaïland since 20 years (Sutantawong et al. 2004). In both programmes, insects are sterilized by irradiation.

SIT impacts the offspring of the target insect. Hence, its success depends on the ability of the released sterile males to mate and inseminate wild females. That is why, all SIT programs follow a quality procedure (FAO 2019) to check the sterile males performance, including sterility, competitiveness, lifespan, flight ability, etc. Sterility and performance are mainly related to the radiation dose: the higher the dose, the higher the sterility but the lower the sterile males performance, and this can be detrimental for SIT operations (Robinson et al. 2002; Lux et al. 2002). Whatever, it is important to consider the best radiation dose such that the sterility is always as close as possible from $100\%$. Sterility is estimated by laboratory experiments where a sample of sterile males are put in cages with fertile females to estimate the percentage of unhatched eggs laid by the sterile mated-females. When the sterile males are $100\%$ sterile, sterile-mated females will lay $100\%$ non-viable (unhatched) eggs. In contrary, when sterile males are not $100\%$ sterile, then sterile-mated females will lay a certain proportion of viable (hatched) eggs, that is called the residual fertility. If this residual fertility is too large, SIT will only have a limited impact on the wild population. Therefore, it is necessary to derive a threshold for the residual fertility in order to insure that below this threshold, SIT is always effective. This threshold may also be helpful to derive the minimal dose of radiation to consider. This issue is even more important when multiple mating occur. Indeed, female fruit flies are polyandrous to increase the genetic diversity in their offspring. However, males have the ability to inhibit females re-mating during a certain amount of time, called the refractory period, before being receptive to mating again (Shelly 2020). In addition some authors show that re-mating increases the fitness of the medfly females (Saul and McCombs 1993; Whittier and Shelly 1993) and the oriental fruit fly females (Shelly 2000; Wei et al. 2015). When it comes to SIT, sperm precedence is important. Indeed, depending on the fruit fly species, the females will use preferably either the sperm of the first mating or the sperm of the second mating. For instance, in Katiyar and Ramirez (1970), it was showed that there is a precedence of the second sperm for medfly females. While, in Zhao et al. (2013), the authors showed that there is a precedence of the first sperm for the oriental fruit fly females.

It is well known that SIT performs better when it is combined with additional control treatments (Pérez-Staples et al. 2021). Here we will consider soil treatment based on the use of soil-entomopathogens that may increase the pupae and adult mortality, and thus reduce the population. One of them, *Beauveria Bassiana*, is naturally present in the soil and used against many pest, including fruit flies (Gava et al. 2020, 2021; Menzler-Hokkanen et al. 2022). The combination of SIT and Enthomopathogenic Fungi (EPF) treatment has been mainly studied by considering sterile males carrying or inoculated by an entomopathogen, such that they become “vectors” of the biocide: this is called the “boosted SIT” (Bouyer and Lefrançois 2014; Diouf et al. 2022; Flores et al. 2013). Wild insects are supposed to be infected through interactions, like mating, with sterile males, such that their biological life cycle is interrupted or their reproductive ability is reduced. Experimental studies, using pyriproxyfen as biocide, have been done or are ongoing in Réunion island, against *aedes spp*. In this paper, since we focus on fruit flies in orchards, we will consider EPFS only. We will show that the combination of EPFS and SIT is beneficial to SIT.

Studying all these combinations and issues in lab, semi-field, or field condition is very difficult, and sometimes impossible. One effective way to address these challenges is through modeling, which allows for theoretical and computational exploration of these problems. Modeling can help identify key threshold parameters relevant to specific issues, aiding field experts in developing optimal release strategies. Since Knipling’s early work (Knipling 1955), a wide variety of SIT models have been developed, starting from simple temporal models to more complex ones, such as sex-structured or stage-structured models, depending on the target pest or vector. Some examples include probabilistic models (Berryman 1967), computational models (Mautner Markhof 1973; Diouf et al. 2022), discrete models (Van den Driessche 1985; Li and Yuan 2015; Barclay 2021), semi-discrete models (Strugarek et al. 2019; Huang et al. 2017; Aronna and Dumont 2020; Dumont and Oliva 2024), and continuous models (Barclay and Mackauer 1980; Esteva and Mo Yang 2005; Anguelov et al. 2012a; Dumont and Tchuenche 2012; Cai et al. 2014; Anguelov et al. 2020; Dumont and Oliva 2024), with some incorporating tools from control theory (Thomé et al. 2010; Bliman et al. 2019; Almeida et al. 2022; Bliman et al. 2024) to optimize the releases strategy of sterile males. Incorporating a spatial component is more challenging, but certain models have addressed this by using partial differential equations (Dufourd and Dumont 2013; Jiang et al. 2014) or adopting a metapopulation approach (Yang et al. 2020; Bliman et al. 2024; Dumont et al. 2024). Note also that boosted SIT models have been developed and studied in Pleydell and Bouyer (2019); Haramboure et al. (2020); Diouf et al. (2022).

In most SIT models, as well as in all “boosted SIT” models, sterile males are always considered $100\%$ sterile. As explained above, this is not the case in reality: there is always residual fertility. This issue is rarely taken into account in SIT models, except in Van den Driessche (1985); Dufourd and Dumont (2013); Aronna and Dumont (2020); Dumont and Oliva (2024); Dumont et al. (2024); Courtois et al. (2025). The issue of re-mating is even less studied in SIT models because, in general, SIT models are applied to mosquitoes that are considered to only mate once. Very few SIT model have been developed for fruit fly and, so far, only one that explicitly takes into account re-mating (Dumont and Oliva 2024).

Indeed, the simultaneous issues of re-mating and residual fertility were recently studied in Dumont and Oliva (2024), showing that the residual fertility threshold beyond which SIT cannot be used, can depend on the refractory period between two mating, and the fitness of double-mated females, first with a wild (sterile) male, and then a sterile(wild) male. In Dumont and Oliva (2024), the authors obtained a generic but complex formula for the residual fertility threshold which encompasses the results obtained in Aronna and Dumont (2020); Van den Driessche (1985). In this manuscript, we will derive a new formula, more compact and more realistic.

In this study, we model the residual fertility differently from Dumont and Oliva (2024), in a more realistic manner because it is closer to the way residual fertility is estimated experimentally (FAO 2019)[Section 6.1, page 60]. As explained above, the residual fertility is the average percentage of hatched eggs deposited by one sterile-mated female. We will explore how residual fertility and re-mating affect SIT efficiency, as well as the critical release rate through continuous or periodic releases. Like in Dumont and Oliva (2024), we consider only single- and double-mating, but our reasoning could be extended to more than two mating. Last but not least, we show that EPFS treatment can relax the constraint on the residual fertility threshold and also reduce the critical release rate threshold.

The outline of the paper is as follows: in Section 1, we build and study a SIT model with continuous release, with re-mating and residual fertility. Then, in section 2, we extend our SIT model to periodic impulsive releases. In section 3, we present and discuss some numerical simulations related to the medfly and the oriental fruit fly, with continuous and periodic releases, with and without EPFS treatment. Finally, we end the paper with a conclusion and several perspectives in section 4.

## The continuous releases SIT model with re-mating and residual fertility.

As with mosquitoes, the life cycle of fruit flies has four distinct stages: egg, larva, pupa and adults. However, their biological cycle is a bit more complex than that of mosquitoes. To summarise, after mating, female fruit flies lay a bunch of fertilised eggs just under the skin of fruit, preferably unripe or semi-ripe fruit. After a couple of days, the eggs hatch and the larvae feed on the fruit until they reach the third and last instar. At this point, they jump to the ground, burrow into the soil and enter the pupae stage. After one to three weeks, the pupae emerge as sexually immature adults. After a couple of days, the males and females become sexually mature and can mate. For the two species of fruit fly considered in this paper, the mating process is quite complex. Males gather in leks ( i.e. male aggregations in mating arenas) of several males(two to twelve), releasing pheromones to attract a female to the lek. The males then court the female, allowing her to compare them and possibly select one for mating.

**Fig. 1:**
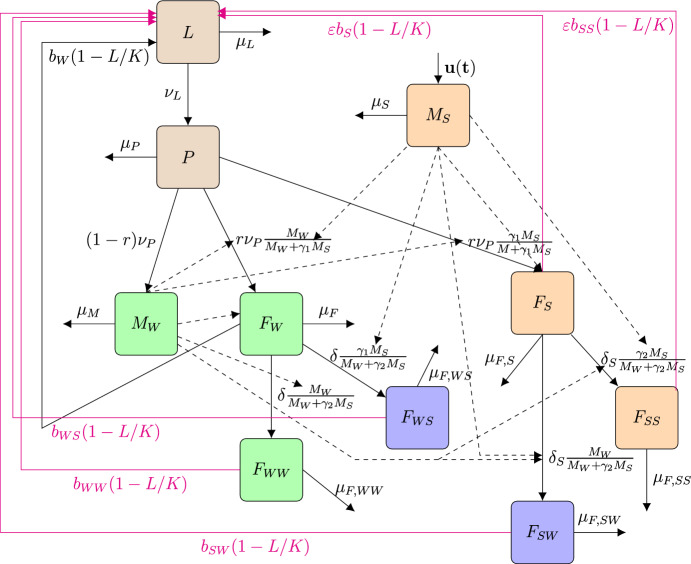
Flow diagram of SIT model (1): the brown squares represents all non-adult stages (Larvae and Pupae); the green squares represent the (fully) wild population (the fertile males and the fertile-mated females only); the blue squares represent the double-mated females (with fertile (sterile) and then sterile (fertile) males); the orange squares represent the sterile population (the released sterile males and the sterile-mated females only). (Color Figure Online)

In the forthcoming model, we will not enter in full biological details, including mating’s details. We only consider the following compartments (see the compartmental diagram in Fig. 1, page 4): the larvae stage, *L*; the pupae stage, *P*; the wild males, $M_W$; the once-mated fertile females, $F_W$; once-mated sterile females, $F_S$; the semi-fertile double-mated females, first by a fertile (sterile) male and then by a sterile (fertile) one, $F_{WS}$ ($F_{SW}$); the double-mated sterile females, $F_{SS}$; the double-mated fertile females, $F_{WW}$; the sterile males, $M_{S}$. While sex determination occurs during embryonic development, we only distinguish males and females at the adult stage in our model, because this is not useful before. We will now describe a little bit some terms that appear in Fig. 1, page 4, and also in the differential system.

We consider the nonlinear oviposition functional (of hatched eggs), $b_*\left( 1-\dfrac{A}{K}\right)$, where $b_*$ is the average oviposition rate for each female compartments, and *K* is the Larvae-carrying capacity of the considered fruit fly host(s). We consider that only hatched eggs enter the larvae compartment. Then, after a certain time, $1/(\nu _L+\mu _L)$ days, they enter the pupae compartment where they stay $1/(\nu _P+\mu _P)$ days before emerging as (sexually mature) individuals that enter either the wild males compartment, $M_W$, at the rate $(1-r)\nu _A$, either the wild (or sterile) females compartment, $F_W$ ($F_S$), at the rate $r\nu _A\dfrac{M_W}{M_W+\gamma _1 M_S}$ ($r\nu _A\dfrac{\gamma _1 M_S}{M_W+\gamma _1 M_S}$), where *r* is the sex-ratio, and $\gamma _1$ represents the competitiveness of the sterile males with respect to the wild males during the first mating. Indeed, released sterile males are supposed to always compete with the wild males in order to mate with females. Thus, the ratio $\dfrac{M_W}{M_W+\gamma _1 M_S}$ ($\dfrac{\gamma _1 M_S}{M_W+\gamma _1 M_S}$), represents the probability for a sexually mature virgin female to mate with a wild (sterile, respectively) male to enter the wild(sterile) single-mated female compartments, $F_W$ ($F_S$). In general, the competitiveness is estimated thanks to a mating experiment between virgin females and wild and sterile males, so that it is considered that the competitiveness is unchanged whatever the number of matings. This is not necessarily true. However, modelling allows us to explore the impact of a competitiveness reduction in the second mating. Indeed very few experiments seem to show that single-mated sterile females have a tendency to re-mate preferably to wild and fertile males. Thus, for the second mating, we assume that the competitiveness parameter is $\gamma _2$, that is less than or equal to $\gamma _1$.

The parameters $\delta$ and $\delta _{S}$ are related to the average refractory periods of females $F_{W}$ and $F_{S}$, respectively. Some studies, like (Kraaijeveld and Chapman 2004), showed that sterile-mated females $F_S$ may have a shorter refractory period, such that $\delta _S\ge \delta \ge 0$. When a female $F_{W}$ re-mates, then she re-mates with either a wild male or a sterile male to enter either the compartment $F_{WW}$ at the rate $\delta \dfrac{M_W}{M_W+\gamma _2 M_S}$, or the compartment $F_{WS}$ at the rate $\delta \dfrac{\gamma _2 M_{S}}{M_W+\gamma _2 M_S}$. This is similar for a sterile female that can re-mate with a wild male and thus enter the compartment $F_{SW}$ at the rate $\delta _{S} \dfrac{M_W}{M_W+\gamma _2 M_S}$ or mates with a sterile male again and enter the compartment $F_{SS}$, at the rate $\delta _S \dfrac{\gamma _2 M_{S}}{M_W+\gamma _2 M_S}$.

As said in the introduction, we will not model the residual fertility, $\varepsilon$, like in Aronna and Dumont (2020); Dumont and Yatat-Djeumen (2022); Dumont and Duprez (2024); Dumont et al. (2024); Dumont and Oliva (2024), i.e. replacing $\dfrac{M_W}{M_W+\gamma M_S}$ by $\dfrac{M_W+\varepsilon M_S}{M_W+\gamma M_S}$. Assuming that sterile males have a certain proportion of fertile sperm, then a female that mates with them once or twice will be able to deposit a daily proportion of viable eggs, $\varepsilon b_S$ or $\varepsilon b_{SS}$. Thus the residual fertility parameters related to $F_S$ and $F_{SS}$ will occur in the birth part of the non-adult stage equation, through $\varepsilon b_S$ and $\varepsilon b_{SS}$. For sake of simplicity, we will consider the same residual fertility parameter, $\varepsilon$. It is important to note that we are not considering residual fertility in the $F_{SW}$ and $F_{WS}$ compartments because these females are still partially fertile. Finally, the parameter $\mu _*$ is the average death rate in each compartment .

In full generality, the release rate, $u(t)\ge 0$, of sterile males may vary in number and in time. We will first consider constant and continuous releases, that is $u(t)\equiv \Lambda$, and derive some results that will be useful later for periodic releases.

The compartmental diagram in Fig 1, page 4, can be translated into the following mathematical model

1 $$\begin{aligned} {\left\{ \begin{array}{ll} \dfrac{dL}{dt}=\left( b_{W}F_W+b_{WW}F_{WW}+b_{SW}F_{SW}+b_{WS}F_{WS}\right. \\ \left. +\varepsilon \left( b_SF_S+b_{SS}F_{SS}\right) \right) \left( 1-\dfrac{L}{K}\right) -\left( \nu _{L}+\mu _{L}\right) L,\\ \dfrac{dP}{dt}= \nu _L L-\left( \nu _{P}+\mu _{P}\right) P, \\ \dfrac{dM_W}{dt}=\left( 1-r\right) \nu _{P}P-\mu _{M}M_W,\\ \dfrac{dF_{W}}{dt}=r\nu _{P}\dfrac{M_W}{M_W+\gamma _1 M_{S}}P-\left( \delta +\mu _{F}\right) F_{W},\\ \dfrac{dF_{S}}{dt}=r\nu _{P}\dfrac{\gamma _1 M_{S}}{M_W+\gamma _1 M_{S}}P-\left( \delta _{S} +\mu _{F,S}\right) F_{S},\\ \dfrac{dF_{WW}}{dt}=\delta \dfrac{M_W}{M_W+\gamma _2 M_{S}}F_{W}-\mu _{F,WW}F_{WW},\\ \dfrac{dF_{WS}}{dt}=\delta \dfrac{\gamma _2 M_{S}}{M_W+\gamma _2 M_{S}}F_{W}-\mu _{F,WS}F_{WS},\\ \dfrac{dF_{SW}}{dt}=\delta _{S} \dfrac{M_W}{M_W+\gamma _2 M_{S}}F_{S}-\mu _{F,SW}F_{SW},\\ \dfrac{dF_{SS}}{dt}=\delta _{S} \dfrac{\gamma _2 M_{S}}{M_W+\gamma _2 M_{S}}F_{S}-\mu _{F,SS}F_{SS},\\ \dfrac{dM_{S}}{dt}=\Lambda -\mu _SM_{S}, \end{array}\right. } \end{aligned}$$

with non-negative initial conditions. Obviously, $M_S$ converges to the following constant equilibrium, $M_S^*=\dfrac{\Lambda }{\mu _S}$. All state variables and parameters of the SIT system (1) are described in Table 7, page 34. Using (Vidyasagar 1980)[Theorem 3.1], it is straightforward to show that studying system (1) is equivalent to study the following system:

2 $$\begin{aligned} \left\{ \begin{array}{l} \dfrac{dL}{dt}=\left( b_{W}F_W+b_{WW}F_{WW}+b_{SW}F_{SW}+b_{WS}F_{WS}\right. \\ \left. +\varepsilon \left( b_SF_S+b_{SS}F_{SS}\right) \right) \left( 1-\dfrac{L}{K}\right) -\left( \nu _{L}+\mu _{L}\right) L,\\ \dfrac{dP}{dt}= \nu _L L-\left( \nu _{P}+\mu _{P}\right) P, \\ \dfrac{dM_W}{dt}=\left( 1-r\right) \nu _{P}P-\mu _{M}M_W,\\ \dfrac{dF_{W}}{dt}=r\nu _{P}\dfrac{M_W}{M_W+\gamma _1 M_{S}^*}P-\left( \delta +\mu _{F}\right) F_{W},\\ \dfrac{dF_{S}}{dt}=r\nu _{P}\dfrac{\gamma _1 M_{S}^*}{M_W+\gamma _1 M_{S}^*}P-\left( \delta _{S} +\mu _{F,S}\right) F_{S},\\ \dfrac{dF_{WW}}{dt}=\delta \dfrac{M_W}{M_W+\gamma _2 M^*_{S}}F_{W}-\mu _{F,WW}F_{WW},\\ \dfrac{dF_{WS}}{dt}=\delta \dfrac{\gamma _2 M^*_{S}}{M_W+\gamma _2 M_{S}}F_{W}-\mu _{F,WS}F_{WS},\\ \dfrac{dF_{SW}}{dt}=\delta _{S} \dfrac{M_W}{M_W+\gamma _2 M^*_{S}}F_{S}-\mu _{F,SW}F_{SW},\\ \dfrac{dF_{SS}}{dt}=\delta _{S} \dfrac{\gamma _2 M^*_{S}}{M_W+\gamma _2 M^*_{S}}F_{S}-\mu _{F,SS}F_{SS}. \end{array} \right. \end{aligned}$$

System (2) is mathematically and biologically well posed: it remains non-negative and bounded.

### Lemma 1.1

For any given, non-negative initial conditions, there exists a unique, non-negative, bounded solution to the Cauchy problem associated with  (2) on $\mathbb {R}_+^9$. It is also continuous and piece-wise continuously differentiable.

It is also straightforward to show that the set $\mathcal {E}_9:=\{ (L,P,M_W,F_W,F_S,F_{WW},F_{WS},F_{SW},F_{SS})^T\in \mathbb {R}_+^9/ L \le K\} \subset \mathbb {R}_+^9$ is forward invariant for system (2), such that any trajectory enters it in finite time.

Without sterile male releases, we can recover the model of the dynamics of the natural/wild pest/vector population from any of systems (1) or (2), with or without EPFS treatment:

3 $$\begin{aligned} \left\{ \begin{array}{l} \dfrac{dL}{dt}=\left( b_WF_{W}+b_{WW}F_{WW}\right) \left( 1-\dfrac{L}{K}\right) -\left( \nu _{L}+\mu _{L}\right) L,\\ \dfrac{dP}{dt}= \nu _L L-\left( \nu _{P}+\mu _{P}\right) P, \\ \dfrac{dM_W}{dt}=\left( 1-r\right) \nu _{P}P-\mu _{M}M_W,\\ \dfrac{dF_{W}}{dt}=r\nu _{P}P-\left( \delta +\mu _{F}\right) F_{W},\\ \dfrac{dF_{WW}}{dt}=\delta F_W-\mu _{F,WW}F_{WW}. \end{array}\right. \end{aligned}$$

Model (3) is almost similar to the one studied in Dumont and Oliva (2024): we define its basic offspring number as follows

4 $$\begin{aligned} \mathcal {R}_W=\mathcal {N}_W \times \left( 1+\dfrac{b_{WW}}{b_W}\dfrac{\delta }{\mu _{F,WW}}\right) , \end{aligned}$$

where

5 $$\begin{aligned} \mathcal {N}_W=\mathcal {N}(b_W,\delta ,\mu _F)=\dfrac{rb_W}{\left( \mu _{F}+\delta \right) }\dfrac{\nu _{L}}{\left( \nu _{L}+\mu _{L}\right) }\dfrac{\nu _{P}}{\left( \nu _{P}+\mu _{P}\right) }. \end{aligned}$$

### Remark 1.2

The parameter $\mathcal {N}_W$ ($\mathcal {R}_W$) represents the average number of (female) offspring that a single-mated female (and a double-mated female) can produce during her life time. It is interesting to note that $\mathcal {N}_W \le \mathcal {R}_W$, so that $\mathcal {N}_W>1$ implies $\mathcal {R}_W>1$, and $\mathcal {R}_W<1$ implies $\mathcal {N}_W<1$.

From Dumont and Oliva (2024), we have the following result:

### Theorem 1.3

System (3) defines a forward dynamical system in $\mathcal {E}_5:= \{ (L,P,M_W,F_W,F_{WW})^T\in \mathbb {R}_+^5/ L \le K\} \subset \mathbb {R}_+^5$. In addition

- if $\mathcal {R}_W{\le }1$, then $\mathbf{{0}}_{\mathbb {R}^5}=(0,0,0,0,0)^T$ is globally asymptotically stable on $\mathcal {E}_5$.
- if $\mathcal {R}_W>1$, then a positive equilibrium $\mathbf{{E}}=(L_0^*,P_0^*,M_0^*,F_{0,W}^*,F_{0,WW}^*)^T$ exists where
  6 $$\begin{aligned} \left\{ \begin{array}{ll} L_0^* =& \left( 1-\frac{1}{\mathcal {R}_W} \right) K, \\ P_0^* =& \dfrac{\nu _{L}}{\left( \nu _{P}+\mu _{P}\right) }\left( 1-\dfrac{1}{\mathcal {R}_W} \right) K, \\ M_0^* =& \dfrac{(1-r)\nu _P}{\mu _M} \dfrac{\nu _{L}}{\left( \nu _{P}+\mu _{P}\right) } \left( 1-\dfrac{1}{\mathcal {R}_W} \right) K, \\ F_{0,W}^* =& \dfrac{r\nu _{P}}{\delta +\mu _{F}} \dfrac{\nu _{L}}{\left( \nu _{P}+\mu _{P}\right) } \left( 1-\dfrac{1}{\mathcal {R}_W} \right) K, \\ F_{0,WW}^{*} =& \dfrac{\delta }{\mu _{F,WW}}\dfrac{r\nu _{A}}{\delta +\mu _{F}} \dfrac{\nu _{L}}{\left( \nu _{P}+\mu _{P}\right) }\left( 1-\dfrac{1}{\mathcal {R}_W} \right) K, \end{array} \right. \end{aligned}$$
  Furthermore, $\textbf{E}$ is globally asymptotically stable on $\mathcal {E}_5 \setminus \left\{ (0,0,M_W,0,0){:} M_W\ge 0\right\}$, while $\mathbf{{0}}_{\mathbb {R}^5}$ is unstable.

For the rest of the paper, we assume that $\mathcal {R}_W>1$. We also define an additional basic Offspring number related to the sterile-mated females, $F_S$ and $F_{SS}$:

7 $$\begin{aligned} \mathcal {R}_S=\mathcal {N}(b_S,\delta _S,\mu _{F,S}) \times \left( 1+\dfrac{b_{SS}}{b_S}\dfrac{\delta _S}{\mu _{F,SS}}\right) . \end{aligned}$$

When $\mathcal {R}_W>1$ and $\Lambda =0$, thanks to Theorem 1.3, the steady state, $\textbf{0}_{\mathbb {R}^9}$, is unstable. However, once sterile males are released, i.e. $\Lambda >0$, it is expected that $\textbf{0}_{\mathbb {R}^9}$ becomes asymptotically stable, at least locally. This is due to a strong Allee effect caused by the release of sterile males (Anguelov et al. 2020). Thanks to this Allee effect, any invading population cannot settle, and, an established population can eventually be eliminated (Anguelov et al. 2020). This is what we will study in the forthcoming results. However, since the control is related to this Allee effect, it means that once SIT is used, it must be maintained. If SIT is perturbed or stopped for any reason, the Allee effect will be lost and the wild population will rise again, more or less rapidly.

Let us show the following result linking the residual fertility, $\varepsilon$, to the existence of a strong Allee effect:

### Lemma 1.4

When $\varepsilon$ is such that $\varepsilon \mathcal {R}_S<1$, or $\varepsilon <\overline{\varepsilon }=1/\mathcal {R}_S$, then $\mathbf{{0}}_{\mathbb {R}^9}$ is always Locally Asymptotically Stable (LAS) for system (2). It is unstable, when $\varepsilon \mathcal {R}_S>1$.

### Proof

see Appendix B, page 35.

From the previous result we deduce that a strong Allee effect only occurs if ${\varepsilon \mathcal {R}_S} <1$. Otherwise, if ${\varepsilon \mathcal {R}_S} > 1$, no Allee effect occurs, meaning that local elimination is impossible, no matter how many sterile males are released. $\square$

### Remark 1.5

Without re-mating, i.e. $\delta _{S}=\delta =0$, in Aronna and Dumont (2020), using a simple model, the authors showed that $\varepsilon$ has to satisfy $\varepsilon \mathcal {N}_{0,W}<1$, where $\mathcal {N}_{0,W}=\mathcal {N}(b_W,0,\mu _W)$, the basic offspring number of the single-mating wild population.

### Remark 1.6

It is also more than interesting to notice that, in fact, this threshold condition, $\varepsilon \mathcal {R}_S<1$, relies only on parameters related to larvae and pupae stages, *L* and *P*, and single- and double-mated sterile females, $F_S$ and $F_{SS}$. In general, the parameters related to $F_{SS}$ are never studied in SIT programs.

### Remark 1.7

EPFS experiments with, for instance, *Beauveria Bassiana*, show an increase in the mortality rates at the pupal and adult stages, and also, sometimes, and the last larval stage (Gava et al. 2020, 2021; Li et al. 2024). Therefore, the higher the mortality rates, the lower $\mathcal {R}_S$, and thus the higher the residual fertility threshold. Therefore we can be confident that EPFS treatment will enhance the efficacy SIT treatment.

### Remark 1.8

Note carefully that the condition on $\varepsilon$ obtained in Lemma 1.4 is more compact, and far less complex than the threshold condition obtained in Dumont and Oliva (2024), $\varepsilon <\varepsilon _{\max }$, where

8 $$\begin{aligned} \varepsilon _{\max }=\frac{2}{\mathcal {N}_W}\dfrac{1}{A\left( \sqrt{1+4\dfrac{B-C}{\mathcal {N}_W b_W A^2}}+1\right) } \end{aligned}$$

where

$$ \begin{array}{l} A=1+\dfrac{\delta _{S}}{\mu _{F,SW}}\dfrac{b_{SW}}{b_{W}}\dfrac{\delta +\mu _{F}}{\delta _{S}+\mu _{F}}+\dfrac{\delta }{\mu _{F,WS}}\dfrac{b_{W,S}}{b_{W}}, \\ B=\delta \left( \dfrac{b_{WW}}{\mu _{F,WW}}-\dfrac{b_{W,S}}{\mu _{F,WS}}\right) , \\ C=\delta _{S}\dfrac{b_{SW}}{\mu _{F,SW}}\dfrac{\delta +\mu _{F}}{\delta _{S}+\mu _{F}}. \end{array} $$

In the previous formula we need all biological parameters related to the partially-sterile females $F_{WS}$ and $F_{SW}$, in addition to those related to the wild mated-females, $F_W$ and $F_{WW}$.

### Remark 1.9

In the case where we may have two distinct residual parameters, $\varepsilon _1$ and $\varepsilon _2$, related to $F_S$ and $F_{SS}$ respectively, then the necessary condition for $\textbf{0}_{\mathbb {R}^9}$ to be LAS becomes

9 $$\begin{aligned} \left( \varepsilon _1+\varepsilon _2\dfrac{b_{SS}\delta _{S}}{b_{S}\mu _{F,SS}}\right) {\mathcal {N}_S}<1. \end{aligned}$$

Altogether, and without any numerical simulations, it is clear that re-mating impact negatively the residual fertility threshold, because $\mathcal {N}_S <\mathcal {R}_S$, such that $\varepsilon<1/\mathcal {R}_S < 1/ \mathcal {N}_S$.

In the following proposition, we show all possible dynamics for system (2), thanks to the sterile male release rate, $\Lambda$. We also show the existence of a release rate threshold, $\Lambda _{cont,\varepsilon }^{crit,*}$ above which, elimination is possible:

### Proposition 1.10

Assume $\mathcal {R}_W>1$, then the following results hold true for system (2):

- Assume $\varepsilon \mathcal {R}_S>1$, then there always exists one positive steady state ${\textbf{E}}^{*}>>0$, whatever the sterile male release rate.
- Assume $\varepsilon \mathcal {R}_S=1$, then, there exists $\gamma \Lambda _{cont,\varepsilon }^{crit,*}>0$, such that
  - If $\Lambda \ge \Lambda _{cont,\varepsilon }^{crit,*}$, there is no positive steady state.
  - If $0\le \Lambda <\Lambda _{cont,\varepsilon }^{crit,*}$, there is one positive steady state $\mathbf{{0}}_{\mathbb {R}^9}<{\textbf{E}}_{*}$.
- Assume $\varepsilon \mathcal {R}_S<1$, then, there exists $\Lambda _{cont,\varepsilon }^{crit} >0$ such that
  - If $\Lambda >\Lambda _{cont,\varepsilon }^{crit}$, there is no positive steady state.
  - If $\Lambda =\Lambda _{cont,\varepsilon }^{crit,}$, there is one positive steady state ${\textbf{E}}_{\varepsilon }^{*}$.
  - If $0<\Lambda <\Lambda _{cont,\varepsilon }^{crit}$, then there are two positive steady states ${\textbf{E}}_{\varepsilon ,-}$ and ${\textbf{E}}_{\varepsilon ,+}$, such that
    $$\begin{aligned} \mathbf{{0}}_{\mathbb {R}^9}<{\textbf{E}}_{\varepsilon ,-}^*<{\textbf{E}}_{\varepsilon ,+}^*. \end{aligned}$$

### Proof

see appendix C, page 36.

In general, it is not possible to derive an explicit formula for $\Lambda _{cont,\varepsilon }^{crit}$, except when $\delta =\delta _S=0$, but it can be estimated numerically by solving equation (16), page 37. $\square$

### Remark 1.11

Notice also that $\Lambda _{cont,\varepsilon }^{crit}$ increases, non-linearly, with respect to $\varepsilon$, as long as $\varepsilon \mathcal {R}_S<1$

### Remark 1.12

It is not possible to prove theoretically the stability properties of the positive equilibria, $E_{\varepsilon ,-}^{*}$ and $E_{\varepsilon ,+}^{*}$, when they exist. Our numerical simulations indicate that $E_{\varepsilon ,-}^{*}$ is unstable while $E_{\varepsilon ,+}^{*}$ is asymptotically stable, and that the equilibria are the only invariant set of the system on $\mathbb {R}^{+}_{9}$.

So far, we know that $\varepsilon \le \varepsilon _{\max }$ ensures elimination only if the wild population is sufficiently small. For practical applications, and, in particular, to ensure that elimination is still possible when the wild population is large, we need to show that the steady-state $\mathbf{{0}}_{\mathbb {R}^9}$ is Globally Asymptotically Stable (GAS).

### Theorem 1.13

Assume $\varepsilon \mathcal {R}_S<1$ and $\Lambda >\Lambda _{cont,\varepsilon }^{crit}$, then $\mathbf{{0}}_{\mathbb {R}^9}$ is GAS for system (2).

### Proof

see appendix D, page 39.

Thanks to the previous results, and like in Dumont and Oliva (2024), we deduce that the residual fertility, $\varepsilon$, is an essential parameter to take into account when designing SIT programs and also to be checked all along the releases. Indeed, if $\varepsilon$ is large, $\Lambda _{cont,\varepsilon }^{crit}$ has to be very large, such that SIT can become ineffective and, thus, fails to reduce the wild population. The classical recommendation is to have the lowest possible residual fertility, with an upper bound depending on the parameters related to single- and double-mating with a sterile male.

Another important aspect is the re-mating of females. A good understanding of this phenomenon can significantly change the constraint on the residual fertility, $\varepsilon$. We will illustrate the impact of re-mating in the numerical section. While continuous releases are very convenient to study from the theoretical point of view, it is more realistic to consider periodic instantaneous (or pulsed) releases. $\square$

## Periodic pulsed SIT releases

In the real, sterile males are not released continuously but periodically and instantaneously, every $\tau$ days. This can be modeled using a semi-discrete approach, like in Anguelov et al. (2020); Aronna and Dumont (2020); Bliman et al. (2019). Thus, in the continuous system (1), it suffices to replace the $M_S$ differential equation and to consider sterile males discrete “events”, to obtain the following impulsive differential system

10 $$\begin{aligned} \left\{ \begin{array}{l} \dfrac{dL}{dt}=\left( b_{W}F_W+b_{WW}F_{WW}+b_{SW}F_{SW}+b_{WS}F_{WS}\right. \\ \left. +\varepsilon \left( b_SF_S+b_{SS}F_{SS}\right) \right) \left( 1-\dfrac{L}{K}\right) -\left( \nu _{L}+\mu _{L}\right) L,\\ \dfrac{dP}{dt}= \nu _L L-\left( \nu _{P}+\mu _{P}\right) P, \\ \dfrac{dM_W}{dt}=\left( 1-r\right) \nu _{P}P-\mu _{M}M_W,\\ \dfrac{dF_{W}}{dt}=r\nu _{P}\dfrac{M_W}{M_W+\gamma _1 M_{S}}P-\left( \delta +\mu _{F}\right) F_{W},\\ \dfrac{dF_{S}}{dt}=r\nu _{P}\dfrac{\gamma _1 M_{S}}{M_W+\gamma _1 M_{S}}P-\left( \delta _{S} +\mu _{F,S}\right) F_{S},\\ \dfrac{dF_{WW}}{dt}=\delta \dfrac{M_W}{M_W+\gamma _2 M_{S}}F_{W}-\mu _{F,WW}F_{WW},\\ \dfrac{dF_{WS}}{dt}=\delta \dfrac{\gamma _2 M_{S}}{M_W+\gamma _2 M_{S}}F_{W}-\mu _{F,WS}F_{WS},\\ \dfrac{dF_{SW}}{dt}=\delta _{S} \dfrac{M_W}{M_W+\gamma _2 M_{S}}F_{S}-\mu _{F,SW}F_{SW},\\ \dfrac{dF_{SS}}{dt}=\delta _{S} \dfrac{ \gamma _2 M_{S}}{M_W+\gamma _2 M_{S}}F_{S}-\mu _{F}F_{SS},\\ \dfrac{dM_{S}}{dt}=-\mu _SM_{S}. \end{array}\right. \end{aligned}$$

with the discrete sterile male releases “events” starting at time $t_S\ge 0$ such that

11 $$\begin{aligned} \left\{ \begin{array}{l} L(t_S+n\tau _{+})=L(t_S+n\tau ),\\ P(t_S+n\tau _{+})=P(t_S+n\tau ),\\ M_W(t_S+n\tau _{+})=M_W(t_S+n\tau ),\\ F_{W}(t_S+n\tau _{+})=F_W(t_S+n\tau ),\\ F_{S}(t_S+n\tau _{+})=F_{S}(t_S+n\tau ),\\ F_{WW}(t_S+n\tau _{+})=F_{WW}(t_S+n\tau ),\\ F_{WS}(t_S+n\tau _{+})=F_{WS}(t_S+n\tau ),\\ F_{SW}(t_S+n\tau _{+})=F_{SW}(t_S+n\tau ),\\ F_{SS}(t_S+n\tau _{+})=F_{SS}(t_S+n\tau ),\\ M_{S}(t_S+n\tau _{+})=M_{S}(t_S+n\tau )+\tau \Lambda _{per}, \qquad n=0,1,2..., \end{array}\right. \end{aligned}$$

and the following non-negative initial conditions

12 $$\begin{aligned} \begin{array}{l} 0\le L(0)=L_0,\quad 0\le P(0)=P_0,\quad 0\le M_W(0)=M_0,\quad 0\le F_W(0)=F_{W,0},\\ 0\le F_{WW}(0)=F_{WW,0}, \\ M_{S}(0)=F_S(0)=F_{WS}(0)=F_{SW}(0)=F_{SS}(0)=0. \end{array} \end{aligned}$$

The right-hand side of system (10)-(11) is locally lipschitz continuous on $\mathbb {R}^{10}$. Thus, using a classic existence theorem Bainov and Simeonov (1995), Theorem 1.1, p. 3, there exist $\beta >0$ and a unique solution defined from $(0,\beta )\rightarrow \mathbb {R}^{10}$ for system (10)-(11)-(12).

Thanks to Eq (10)$_{{10}}$, with the pulsed event defined in Eq (11)$_{{10}}$, it is straightforward to show that, as $t\rightarrow +\infty$, $M_{S}$ converges toward the periodic solution

$$ M_{S,per}(t)={\displaystyle \frac{\tau \Lambda _{per}}{1-e^{-\mu _S\tau }}e^{-\mu _S(t-\lfloor t/\tau \rfloor \tau )}}, $$

such that the long time dynamics of system (10)-(11) can be deduced by studying

13 $$\begin{aligned} \left\{ \begin{array}{l} \dfrac{dL}{dt}=\left( b_{W}F_W+b_{WW}F_{WW}+b_{SW}F_{SW}+b_{WS}F_{WS}\right. \\ \left. +\varepsilon \left( b_SF_S+b_{SS}F_{SS}\right) \right) \left( 1-\dfrac{L}{K}\right) -\left( \nu _{L}+\mu _{L}\right) L,\\ \dfrac{dP}{dt}= \nu _L L-\left( \nu _{P}+\mu _{P}\right) P, \\ \dfrac{dM_W}{dt}=\left( 1-r\right) \nu _{P}P-\mu _{M}M_W,\\ \dfrac{dF_{W}}{dt}=r\nu _{P}\dfrac{M_W}{M_W+\gamma _1 M_{S,per}}P-\left( \delta +\mu _{F}\right) F_{W},\\ \dfrac{dF_{S}}{dt}=r\nu _{P}\dfrac{\gamma _1 M_{S,per}}{M_W+\gamma _1 M_{S,per}}P-\left( \delta _{S} +\mu _{F,S}\right) F_{S},\\ \dfrac{dF_{WW}}{dt}=\delta \dfrac{M_W}{M_W+\gamma _2 M_{S,per}}F_{W}-\mu _{F,WW}F_{WW},\\ \dfrac{dF_{WS}}{dt}=\delta \dfrac{\gamma _2 M_{S,per}}{M_W+\gamma _2 M_{S,per}}F_{W}-\mu _{F,WS}F_{WS},\\ \dfrac{dF_{SW}}{dt}=\delta _{S} \dfrac{M_W}{M_W+\gamma _2 M_{S,per}}F_{S}-\mu _{F,SW}F_{SW},\\ \dfrac{dF_{SS}}{dt}=\delta _{S} \dfrac{ \gamma _2 M_{S,per}}{M_W+\gamma _2 M_{S,per}}F_{S}-\mu _{F}F_{SS}. \end{array}\right. \end{aligned}$$

Setting

$$ \left\{ \begin{array}{l} {\overline{M}}_{S}=\max \limits _{t\in [0,\tau ]}M_{S}^{per}(t)={\displaystyle \frac{\tau \Lambda _{per}}{1-e^{-\mu _S\tau }},} \\ {\underline{M}}_{S}=\min \limits _{t\in [0,\tau ]}M_{S}^{per}(t)={\displaystyle \frac{\tau \Lambda _{per}}{1-e^{-\mu _S\tau }}e^{-\mu _S\tau }={\overline{M}}_{S}e^{-\mu _S\tau },} \end{array}\right. $$

it is obvious to deduce that, for *t* sufficiently large, system (13) is lower and upper bounded by the following systems

$$ (U)\left\{ \begin{array}{l} \dfrac{dL}{dt}=\left( b_{W}F_W+b_{WW}F_{WW}+b_{SW}F_{SW}+b_{WS}F_{WS}\right. \\ \left. +\varepsilon \left( b_SF_S+b_{SS}F_{SS}\right) \right) \left( 1-\dfrac{L}{K}\right) -\left( \nu _{L}+\mu _{L}\right) L,\\ \dfrac{dP}{dt}= \nu _L L-\left( \nu _{P}+\mu _{P}\right) P, \\ \dfrac{dM_W}{dt}=\left( 1-r\right) \nu _{P}P-\mu _{M}M_W,\\ \dfrac{dF_{W}}{dt}=r\nu _{P}\dfrac{M_W}{M_W+{\underline{M}}_{S}}P-\left( \delta +\mu _{F}\right) F_{W},\\ \dfrac{dF_{S}}{dt}=r\nu _{P}P-\left( \delta _{S} +\mu _{F,S}\right) F_{S},\\ \dfrac{dF_{WW}}{dt}=\delta \dfrac{M_W}{M_W+\gamma _{2}{\underline{M}}_{S}}F_{W}-\mu _{F,WW}F_{WW},\\ \dfrac{dF_{WS}}{dt}=\delta F_{W}-\mu _{F,WS}F_{WS},\\ \dfrac{dF_{SW}}{dt}=\delta _{S}\dfrac{M_W}{M_W+\gamma _{2}{\underline{M}}_{S}}F_{S}-\mu _{F,SW}F_{SW},\\ \dfrac{dF_{SS}}{dt}=\delta _{S}F_S-\mu _{F,SS}F_{SS}, \end{array}\right. $$

$$ (L)\left\{ \begin{array}{l} \dfrac{dL}{dt}=\left( b_{W}F_W+b_{WW}F_{WW}+b_{SW}F_{SW}+b_{WS}F_{WS}\right. \\ \left. +\varepsilon \left( b_SF_S+b_{SS}F_{SS}\right) \right) \left( 1-\dfrac{L}{K}\right) -\left( \nu _{L}+\mu _{L}\right) L,\\ \dfrac{dP}{dt}= \nu _L L-\left( \nu _{P}+\mu _{P}\right) P, \\ \dfrac{dM_W}{dt}=\left( 1-r\right) \nu _{P}P-\mu _{M}M_W,\\ \dfrac{dF_{W}}{dt}=r\nu _{P}\dfrac{M_W}{M_W+\gamma _1 {\overline{M}}_{S}}P-\left( \delta +\mu _{F}\right) F_{W},\\ \dfrac{dF_{S}}{dt}=r\nu _{P}\dfrac{\gamma _1 e^{-\mu _{S}\tau }{\overline{M}}_{S}}{M_W+\gamma _1 e^{-\mu _{S}\tau }{\overline{M}}_{S}}P-\left( \delta _{S} +\mu _{F,S}\right) F_{S},\\ \dfrac{dF_{WW}}{dt}=\delta \dfrac{M_W}{M_W+\gamma _{2}{\overline{M}}_{S}}F_{W}-\mu _{F,WW}F_{WW},\\ \dfrac{dF_{WS}}{dt}=\delta \dfrac{\gamma _{2}e^{-\mu _{S}\tau }{\overline{M}}_{S}}{M_W+\gamma _{2}e^{-\mu _{S}\tau }{\overline{M}}_{S}}F_{W}-\mu _{F,WS}F_{WS},\\ \dfrac{dF_{SW}}{dt}=\delta _{S}\dfrac{M_W}{M_W+\gamma _{2}{\overline{M}}_{S}}F_{S}-\mu _{F,SW}F_{SW},\\ \dfrac{dF_{SS}}{dt}=\delta _{S}\dfrac{\gamma _{2}e^{-\mu _{S}\tau }{\overline{M}}_{S}}{M_W+\gamma _{2}e^{-\mu _{S}\tau }{\overline{M}}_{S}}F_{S}-\mu _{F,SS}F_{SS}. \end{array}\right. $$

Note also that system (U) is similar to system (17), used in appendix D, except that $M_S^*$ is replaced by ${\overline{M}}_{S}$. First of all, it is important to show the following result

### Lemma 2.1

When $\varepsilon \mathcal {R}_S<1$, the steady state $\textbf{0}_{\mathbb {R}^9}$ is always Locally Asymptotically Stable (LAS) for systems (L) and (U). It is unstable, when $\varepsilon \mathcal {R}_S>1$.

### Proof

See appendix E, page 40. $\square$

The previous Lemma guarantees that $\textbf{0}_{\mathbb {R}^9}$ is also locally asymptotically stable for the periodic system (13) when $\mathcal {R}_s\varepsilon <1$. Then, we can apply Proposition 1.10, page 8, to system (U)

### Proposition 2.2

Assume $\varepsilon \mathcal {R}_S <1$. There exists a positive constant $\Lambda ^{U,crit}_{cont,\varepsilon }$, such that when $\Lambda _{per}>\dfrac{e^{\mu _S\tau }-1}{\tau \mu _S}\Lambda ^{U,crit}_{cont,\varepsilon }$, the steady state $\textbf{0}_{\mathbb {R}^{9}}$ is Globally Asymptotically Stable for system (U) and, thus, for the periodic system (13).

From Proposition 2.2, we can deduce the existence of a periodic critical release rate $\Lambda _{per,\varepsilon }^{crit}>0$ such that

$$ \dfrac{1-e^{-\mu _S\tau }}{\tau \mu _S} \Lambda ^{crit}_{cont,\varepsilon } \le \Lambda _{per,\varepsilon }^{crit} \le \dfrac{e^{\mu _S\tau }-1}{\tau \mu _S}\Lambda ^{crit}_{cont,\varepsilon }. $$

When $\tau$ goes to 0, i.e. the time between 2 consecutive releases is going to 0, we have

$$ \dfrac{\left( e^{\mu _S\tau }-1\right) \Lambda ^{crit}_{cont,\varepsilon }}{\tau \mu _S} \longrightarrow \Lambda ^{crit}_{cont,\varepsilon }, $$

and

$$ \dfrac{\left( 1-e^{-\mu _S\tau }\right) \Lambda ^{crit}_{cont,\varepsilon }}{\tau \mu _S} \longrightarrow \Lambda ^{crit}_{cont,\varepsilon }, $$

such that we recover the result obtained for continuous releases in Proposition 1.10, page 8, that is

$$ \Lambda _{per,\varepsilon }^{crit} \longrightarrow _{\tau \rightarrow 0} \Lambda ^{crit}_{cont,\varepsilon }. $$

### Proposition 2.3

When $\varepsilon \mathcal {R}_S <1$, and $0<\tau \Lambda _{per} \le \dfrac{\left( 1-e^{-\mu _S\tau }\right) }{\mu _S}\Lambda ^{crit}_{cont,\varepsilon }$, the set

$$ \begin{array}{ll} \Omega _9=& \left\{ (L,P,M_W,F_W,F_S,F_{WW},F_{WS},F_{SW},F_{SS})^T\in \mathbb {R}_+^9: \right. \\ \\ & \left. {\overline{E}}_{1,9D} \le (L,P,M_W,F_W,F_S,F_{WW},F_{WS},F_{SW},F_{SS}) \le \textbf{E} \right\} \end{array} $$

is positively invariant by system (13), where $\textbf{E}$, the initial wild equilibrium, is defined in Theorem 1.3, page 7.

Finally, using the previous result and Brouwer fixed point theorem, with comparison arguments, it is possible to show

### Theorem 2.4

Assume $\mathcal {R}_W>1$, $\varepsilon \mathcal {R}_S<1$, and $0<\tau \Lambda _{per} \le \dfrac{\left( 1-e^{-\mu _S\tau }\right) }{\mu _S}\Lambda ^{crit}_{cont,\varepsilon }$. Then, for each initial condition in $\Omega _9$, system (13) has at least one positive $\tau$-periodic solution ${\textbf{E}}_{per}$ such that ${\textbf{E}}_{per} \in \Omega _9$.

It is not possible to find an analytical formula for $\Lambda _{per,\varepsilon }^{crit}$. However, it is possible to estimate it numerically, by solving system (10)-(11)-(12), page 9, using an iterative approach. When $\tau$ goes to 0, meaning that the periodic releases become continuous, $\Lambda _{per,\varepsilon }^{crit}$ converges to $\Lambda ^{crit}_{cont,\varepsilon }$.

## Numerical simulations

We now apply the previous theoretical results to the Mediterranean fruit fly, *Ceratis capitata*, using the parameter values given in Dumont and Oliva (2024) in order to compare our results with those of Dumont and Oliva (2024). We also consider the oriental fruit fly, *Bactrocera dorsalis*, using the parameter values from Diouf et al. (2022), and also related to an ongoing SIT control program, AttracTIS, in Réunion island.

Thanks to Appendix C and equation (16), we will derive estimates of $\Lambda _{cont,\varepsilon }^{crit}$, the critical release rate for continuous releases, and, thus, highlight the impact of residual fertility, re-mating, and competitiveness, with and without EPFS treatment, on SIT control treatment. In particular, $M_0^*$ being the size of the male population at equilibrium defined in (6)$_3$, we will estimate $\dfrac{\Lambda _{cont,\varepsilon }^{crit}}{M_{0}^{*}}$, the critical release ratio that is, in general, considered in operational SIT programs. Similarly, we will also estimate the critical periodic release ratio, $\dfrac{\tau \Lambda _{cont,\varepsilon }^{crit}}{M_{0}^{*}}$, by solving directly system (10), page 9.

### Medfly parameters estimate

Most of parameter values for the Mediterranean fruit fly come mainly from Dumont and Oliva (2024), where the authors consider numerous references to derive some biological parameters obtained from the literature for Mediterranean fruit fly reared on peaches, and on sterile males from the V8 GSS strain (Vienna-8 Genetic Sexing Strain). We also put additional values for the biological parameters related to the Larvae and Pupae stages. See Table 1, page 13.

**Table 1:** Some parameters values for *C. capitata* available in the literature (Dumont and Oliva 2024)

Parameter	Numerical values	Unit	References
$b_W$	12.1348	day$^{-1}$	(Ghabbari and Ben Jemâa 2022)
$\nu _L$	0.07431	day$^{-1}$	(Ghabbari and Ben Jemâa 2022)
$\mu _L$	0.032872	day$^{-1}$	(Ghabbari and Ben Jemâa 2022)
$\nu _P$	0.107961	day$^{-1}$	(Ghabbari and Ben Jemâa 2022)
$\mu _P$ $\mu _{P,EPF}$	0.0052888 0.04492639	day$^{-1}$	(Ghabbari and Ben Jemâa 2022, 2022; Gava et al. 2021)
*r*	0.53	-	(Ghabbari and Ben Jemâa 2022)
$\mu _F$ $\mu _{F,EPF}$	1/42.66 1/36.15	day$^{-1}$	(Ghabbari and Ben Jemâa 2022, 2022; Gava et al. 2021)
$\mu _M$ $\mu _{M,EPF}$	1/50.33 1/42.65	day$^{-1}$	(Ghabbari and Ben Jemâa 2022, 2022; Gava et al. 2021)
$\mu _{F,WW}$	$0.7941176 \times \mu _F$	day$^{-1}$	(Whittier and Shelly 1993; Saul and McCombs 1993)
$b_{WW}$	$1.29705 \times b_W$	day$^{-1}$	(Whittier and Shelly 1993)
$\delta$	0.16	day$^{-1}$	(Morelli et al. 2013)
$\mu _S$	0.2310	day$^{-1}$	(Plant and Cunningham 1991)
$\gamma$ $\gamma _{GRO}$	0.6129 2.03	-	RSI = $0.34\pm 0.004$ RSI = 0.67 (Paranhos et al. 2010)
$\delta _S$ $\delta _{S,GRO}$	0.3161 0.2361	day$^{-1}$	(Morelli et al. 2013)

As expected, re-mating has a significant impact on the fitness of Medfly females: see (Saul and McCombs 1993; Whittier and Shelly 1993; Lee et al. 2003). Unfortunately, re-mating is complex to study, in particular when the focus is on the combination between wild mating and sterile mating, taking into account the order of re-mating, that can impact the fertility of double-mated females. Only very few numerical values are available or can be estimated for $\delta$, $\delta _S$, $b_{WS}$ and $b_{SW}$ and $\mu _{WW}$ (Morelli et al. 2013; Katiyar and Ramirez 1970; Lee et al. 2003; Whittier and Shelly 1993), but not for others, like $\mu _{WS}$ and $\mu _{SW}$. In Dumont and Oliva (2024), the authors assumed that sterile females have the same death-rate than wild females, that is $\mu _{WS}=\mu _{SW}=\mu _{F,S}$, which seems to be a reasonable assumption. For parameters $b_{WS}$ and $b_{SW}$, two major parameters in the whole dynamics, we will consider two cases.

Contrary to Dumont and Oliva (2024), where the variables $F_S$ and $F_{SS}$ were not important in the dynamics, they are now important as they participate in the birth-rate functional in system (2). Thus, we have 4 parameters, namely $b_S$, $b_{SS}$, $\mu _{F,S}$ and $\mu _{F,SS}$ that may play an important role in the dynamics of the system and also on the estimate of $\mathcal {R}_{S}$. According to Katiyar and Ramirez (1970)[Table 3], it seems that wild-mated and sterile-mated females can deposit the same amount of eggs, such that $b_S=b_W$. Re-mating has a positive impact on the fitness of the medfly: $\mu _{WW}=\dfrac{27}{34}\mu _W$ and $b_{WW}=\dfrac{4.1765}{3.22}b_W$. Unfortunately, there is no data related to the double sterile-mated females, such that we will assume that, like for the double-mated wild females, we have $b_{SS}=\dfrac{4.1765}{3.22}\times b_S$ (a double-mated sterile female can deposit more eggs than a single-mated sterile female). Similarly, we will consider that $\mu _{F,SW}=\mu _{F,SW}=\mu _{F,S}=\mu _F$, and $\mu _{SS}=\mu _{WW}$.

Last, in order to increase the competitiveness of the sterile males, several treatments have been developed: for *C. capitata*, this is the addition of ginger root oil (GRO) (Shelly and McInnis 2001; Paranhos et al. 2013). Thus we will consider a competitiveness parameter without and with GRO- treatment, i.e. $\gamma$ and $\gamma _{GRO}$.

We will also consider SIT-EPFS combination. In particular, we consider experimental results with the fungus *Beauveria Bassiana* because it is used on a wide range of pest insects, including fruit flies, in many agricultural and horticultural crops. It was also showed some *B. Bassiana* strains can be highly virulent against *Ceratitis capitata*. From Gava et al. (2021), we derive that the pupae mortality increases between $32\%$ and $38.6\%$, that is, on average, $35\%$. Similarly, the adult mortality increases between 14.6 and $22.5\%$ Gava et al. (2021), that is, on average, $18\%$. This will necessarily impacts the basic offspring numbers, $\mathcal {R}_W$ and $\mathcal {R}_S$, and, then, the residual fertility threshold, and the critical release rates, $\Lambda _{\varepsilon ,cont}^{crit}$ and $\Lambda _{\varepsilon ,per}^{crit}$. In order to maintain a sufficient and constant amount of EPF in the soil, and thus to ensure a constant additional mortality, we simply consider that EPF can be sprayed onto the ground via the irrigation system, and, then, spread through the soil via the water.

### Oriental fruit fly parameters estimate

For *B. dorsalis*, data and parameters values are more difficult to find because they are fewer SIT programs targeting the oriental fruit fly. However, we will consider the parameters values given in Ekesi et al. (2006) on Mango fruits. It was also showed that sterile males treated with Methyl-Eugenol(ME) enhance their competitiveness. In Shelly et al. (2010); Shelly and McInnis (2015); Orankanok et al. (2013); Ji et al. (2013), biologists got estimates of the Relative Sterility Index (RSI), with and without ME-treatment, from which we can derive the competitiveness index, $\gamma$. In general, ME exposure also increase the RSI compared to non-exposure. Thus, thanks in particular to Ji et al. (2013)[Table 1], without ME-treatment, the mean RSI is low, around 0.28, leading to $\gamma =0.39$. When sterile males are treated with ME, then according to Ji et al. (2013)[Table 1], the competitiveness index increases such that $\gamma =0.39\times 2.63 \approx 1.0257$, on average. According to Shelly (2000) there were no differences in female survival, fecundity or fertility between females that mated with ME-treated males or not-treated males. In addition, the impact of a sterile male on re-mating and refractory period is also studied in Ji et al. (2013); Shelly (2020): the authors indicated that re-mating for a sterile-mated female occurs more rapidly than for a wild-mated female, i.e $\delta _S>\delta$. However, there is no real estimate of the refractory periods. The readers have to be aware that these kind of experiments are long and tedious and that is why there are not so many data available. As part of the GEMDOTIS and the ATTRACTIS projects, the lifespan of sterile fruit flies, irradiated at 80 Gy, was estimated to be half that wild insects, at least in laboratory conditions. That is why we will consider $\mu _S={2 \times } \mu _M$ in the simulations.

Also, like the medfly, multiple mating increase the fitness of the oriental fruit fly (Shelly 2000; Wei et al. 2015). In particular, more offspring are produced, i.e. $b_{WW}>b_W$. In Shelly (2000), multiple-mated females laid significantly more eggs that singly mated females: on average the increase is around $\dfrac{628}{488}\approx 1.287$, such that we can deduce that $b_{WW}=1.287\times b_W$. Like for *C. capitata*, there is no data for double sterile-mated females, we assume that $b_{SS}=b_{WW}$. Since there is no information about the death rates, we will also consider that $\mu _{F,SW}=\mu _{F,SW}=\mu _{F,S}=\mu _F$, and $\mu _{SS}=\mu _{WW}$.

For *B. dorsalis*, the mean refractory period is significant: 20.5 days (Wei et al. 2015; Diouf et al. 2022), on average. Thanks to Shelly (2000, 2020), almost $52\%$ of the females re-mate. In Shelly (2020), the author compared the re-mating of females with initial mating either with a wild male or a sterile male. There, the sterile male comes from a genetic sexing strain, that allows to separate easily male pupae from female pupae. The level of irradiation is 100 Gray. One of the main result is that sterile males increase the proportion of females, $F_S$, that re-mate: it becomes $72.8\%$, on average. Thus, clearly, we are in the case where $\delta _{S}>\delta$. When sterile males are ME-treated, they have a better competitiveness but they also increase the proportion of sterile-mated that re-mate by $33.2\%$ thanks to Ji et al. (2013)[Table 3], such that, by induction, $\delta _{S,ME}>\delta _S>\delta$.

**Table 2:** Parameters values for *B. dorsalis*

**Parameter**	Numerical values	Unit	References
$b_W$	12.49	day$^{-1}$	(Ekesi et al. 2006)
$\nu _L$	0.06811	day$^{-1}$	
$\mu _L$	0.02198	day$^{-1}$	(Ekesi et al. 2006)
$\nu _P$	0.06524	day$^{-1}$	(Ekesi et al. 2006)
$\mu _P$ $\mu _{P,EPF}$	0.015403 0.0175656	day$^{-1}$	(Ekesi et al. 2006, 2006; Wang et al. 2021)
*r*	0.5059	-	
$\mu _F$ $\mu _{F,EPF}$	1/75.1 1/43.46	day$^{-1}$	(Ekesi et al. 2006) (Ekesi et al. 2006; Wang et al. 2021)
$\mu _M$ $\mu _{M,EPF}$	1/86.4 1/50	day$^{-1}$	(Ekesi et al. 2006) (Ekesi et al. 2006; Wang et al. 2021)
$\mu _{F,WW}$	$\mu _F$	day$^{-1}$	-
$b_{WW}$	$1.287 \times b_W$	day$^{-1}$	(Shelly 2000)
$\delta$	0.52/20.5	day$^{-1}$	(Shelly 2020)
$\mu _S$	2/86.4	day$^{-1}$	-
$\gamma$ $\gamma _{ME}$	0.39 1.0257	-	(Ji et al. 2013)[Table 1]
$\delta _S$ $\delta _{S,ME}$	0.728/20.5 0.95904/20.5	day$^{-1}$	(Shelly 2020)

From Bertin et al. (2010), for *C. capitata*, there is a tendency of second sperm precedence, at least for the first ovipositions, and then it decreases in favor of the first sperm. From Zhao et al. (2013), for *B. dorsalis*, this is the contrary: there is a tendency of first sperm precedence. Indeed, if the total amount of laid eggs is (statistically) similar for $F_{WS}$ and $F_{SW}$, the proportion of eggs hatched is not: it is $71.7\%$ for $F_{WS}$ and $54.9\%$ for $F_{SW}$. It is important to note that this result confirms that the first sperm seems to have the precedence to the second sperms since $b_{WS}>b_{SW}$, at least for *B. dorsalis*.

However, it would be more than welcome to conduct experiments, like (Zhao et al. 2013), to clearly estimate the total amount of hatching eggs laid by $F_{WS}$, $F_{SW}$, $F_{WW}$, also $F_{SS}$, as well as their mean lifespan, to estimate the eggs deposit rates and the death rates for these compartments. In general, these parameters are studied in all SIT programs, as part of the quality control, but for $F_S$ only.

The tolerable value for residual fertility is very low according to the chosen parameters values. This would give reason to SIT implementation programs that choose a fully sterilizing dose, such as Abraham et al. (2021).

Like for *C. capitata*, we will consider the combination between SIT and EPFS treatment, considering *Beauveria Bassiana* strains. From Wang et al. (2021), we know that one *B. bassiana* strain could be very efficient, increasing the daily mortality rates for the pupae and adult stages, by $14.04\%$ and $72.8\%$, such that $\mu _{P,EPFS}\approx 0.0175656$, $\mu _{F,EPFS} \approx 1/43.46$, and $\mu _{M,EPFS} \approx 1/50$. It is important to notice that the additional pupal mortality due to *B. Bassiana* may change according to the type of soil in which the the pupation occurs (Menzler-Hokkanen et al. 2022).

Numerous numerical simulations could be performed. That is why, for the future simulations of both biological models, we will only consider two main cases.

- Without re-mating, i.e. $\delta =\delta _S=0$: females mate only once whether the male is wild or sterile. This is what is usually (and implicitly) assumed in most of the SIT models.
- With re-mating, such that $0<\delta \le \delta _S$. This is supposed to be the “standard” case for both fruit flies: after mating with a sterile male, a female may re-mate faster, or as fast, than a female that mated with a wild male.

Within the two previous cases, will also consider two sub-cases for the competitiveness.

- Equal competitiveness, i.e. $\gamma _2=\gamma _1$: whatever the first or the second mating, a sterile male keep the same competitiveness
- Lost of competitiveness, i.e. $\gamma _2<\gamma _1$: fruit flies females have a tendency to re-mate preferentially with a wild males rather than with a sterile male, such that we suppose that sterile males are less competitive for the second mating.

We also consider different numerical values for $b_{WS}$ and $b_{SW}$ for continuous and periodic releases to illustrate the importance of the double-mated females parameters. In all simulations, SIT-treatment starts at time $t=100$ days.

### Numerical simulations: *C. capitata*

We now derive some simulations related to simulations done in Dumont and Oliva (2024). In particular, we will consider two cases, namely $b_{WS}=b_{SW}=0.5 \times b_{W}$ meaning that there is a random choice of the double-mated females to use either the fertile or sterile sperm. Then, taking into account the second sperm preference, we will consider the case where $b_{WS}<b_{SW}$, thanks to Lee et al. (2003), where $b_{WS}=0.1532 \times b_W$ and $b_{SW}=0.65 \times b_W$.

What is very important to understand here is that $1/\mathcal {R}_S$, the residual fertility threshold only depends on the single- and double-mated sterile females parameters. Thus, if one or several parameters related to the sterile females may change, this will impact the residual fertility threshold, $1/\mathcal {R}_{S}$.

Thanks to the parameters values given in Table 1, we find that $\mathcal {N}_{W}\approx 181.3368$ and $\mathcal {R}_W \approx 288.25$ (285.81) without (with) GRO-treatment. As expected $\mathcal {R}_W>\mathcal {N}_W$, showing that re-mating can increase the amount of offspring by almost $60\%$. Thanks to the sterile insect parameters, we derive Table 3, page 15, where the values for the sterile population basic offspring number, with re-mating, $\mathcal {R}_S$, without re-mating, $\mathcal {N}_S$, are computed with and without GRO-treatment. Since we have considered that sterile females are similar to wild females, we have $\mathcal {N}_S=\mathcal {N}_W$ and $R_S=R_W$. That is why, with or without GRO-treatment, it is important to study the mating and re-mating behaviour of females that mated either with wild males or sterile males.

**Table 3:** SIT only - *C. capitata* sterile insect’s Basic Offspring numbers and residual fertility thresholds, with and without GRO treatment

SIT	$\mathcal {N}_{S}$	$\overline{\varepsilon }$	$\mathcal {R}_{S}$	$\overline{\varepsilon }$
SM	181.3368	0.0055146	288.2504	0.00349884
SM-GRO	181.3368	0.0055146	285.8088	0.0034972

Then, obviously, without mating, the constrained on the residual fertility becomes $\varepsilon <\overline{\varepsilon }=1/\mathcal {N}_{S} \approx 0.0055146$, while with double-mating we have $\varepsilon <\overline{\varepsilon }=1/\mathcal {R}_S \approx 0.00349884$ (without GRO-treatment) or $\varepsilon <\overline{\varepsilon }=1/\mathcal {R}_S \approx 0.0034972$ (with GRO-treatment). Thus clearly, there is an impact of re-mating on the admissible residual fertility threshold. Note also that we obtain almost similar results for the residual fertility thresholds than those obtained in Dumont and Oliva (2024)[Table 5]. However, these thresholds are now fixed and cannot change like it was the case in Dumont and Oliva (2024)(compare Table 5 and Table 6), when $b_{SW}$ and $b_{WS}$ change and if re-mating occurs or not. From a practical point of view, this is better.

The SIT - EPFS combination leads to a clear improvements in the residual fertility threshold values. Indeed, the basic offspring without and with re-mating decay substantially (see the second and fourth columns in Table 4 and compare them to Table 3), such that the constraints on the residual fertility are relaxed (see the third and fifth columns in Table 4 and compare them to Table 3). Thus, from that point of view the combination SIT-EPFS is clearly beneficial.

**Table 4:** SIT-EPFS combination - *C. capitata* sterile insect’s Basic Offspring numbers and residual fertility thresholds, with and without GRO treatment.

SIT-EPF	$\mathcal {N}_{S}$	$\overline{\varepsilon }$	$\mathcal {R}_{S}$	$\overline{\varepsilon }$
SM	113.832	$<0.008784876$	180.1217	$<0.005551801$
SM-GRO	113.832	$<0.008784876$	178.364	$<0.005606513$

Now we derive simulations related to the releases rate threshold for continuous and periodic releases.

**Fig. 2:**
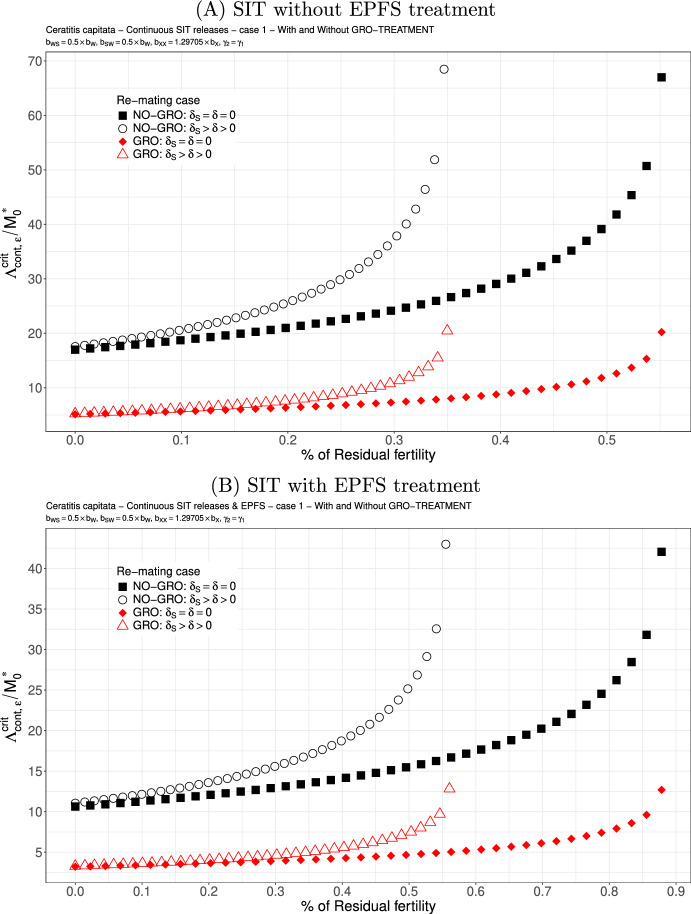
SIT Treatment against *Ceratitis capitata* with equal competitiveness, $\gamma _2=\gamma _1$ - Critical release ratio for continuous releases as a function of residual fertility - re-mating case 1 with $b_{W,S}=b_{S,W}=0.5 \times b_{W}$. Simulations without **(A)** and with EPFS treatment **(B)**, and different re-mating configurations: the black squares, the NO re-mating case without GRO-treatment; the black circles, the re-mating case without GRO-treatment; the red losanges, the NO re-mating case with GRO-treatment; the red triangles, the re-mating case with GRO-treatment. (Color Figure Online)

**Fig. 3:**
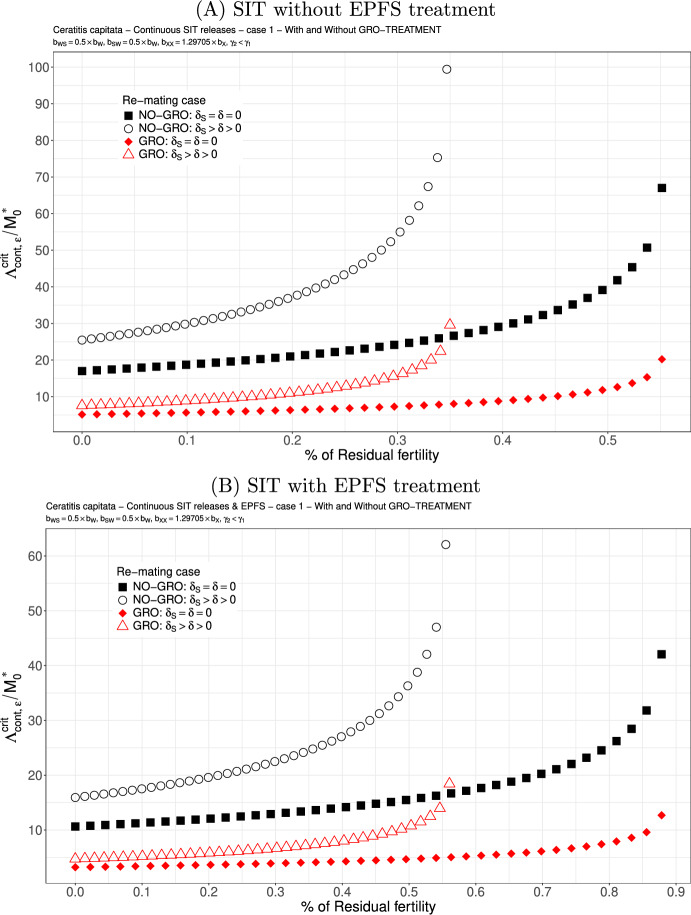
SIT Treatment against *Ceratitis capitata* with non-equal competitiveness, $\gamma _2=0.5\times \gamma _1$ - Critical release ratio for continuous releases as a function of residual fertility - re-mating case 1 with $b_{W,S}=b_{S,W}=0.5 \times b_{W}$. Simulations without **(A)** and with EPFS treatment **(B)**, and different re-mating configurations: the black squares, the NO re-mating case without GRO-treatment; the black circles, the re-mating case without GRO-treatment; the red losanges, the NO re-mating case with GRO-treatment; the red triangles, the re-mating case with GRO-treatment. (Color Figure Online)

1. We assume that $b_{WS}=b_{SW}=0.5 \times b_{W}$: see Figs. 2 and 3. We also observe that the sterile males releases threshold increases with $\varepsilon$ until it reaches one of the residual fertility thresholds, either $1/\mathcal {N}_S$ (without re-mating) or $1/\mathcal {R}_S$ (with mating), estimated in Table 3, page 15. The higher the residual fertility, the higher the release threshold. However, the threshold values become (very) large when they are close from the residual fertility threshold. As expected (Barry et al. 2003), the GRO-treatment, which mainly improves the competitiveness, has a positive impact on the releases rate without and with re-mating. When residual fertility is low, the release rate thresholds, with and without re-mating, are almost similar, especially when GRO-treatment is considered. For instance, when the residual fertility is less than 0.25 (or 0.55 with EPFS), then releasing $10\times M_0^*$ males every day is sufficient to decay the population to 0. However, a comparison of Fig. 2(A) and Fig. 2(B) clearly shows an improvement when the SIT-EPFS combination is used, in terms of both the release ratio and the residual fertility thresholds. Finally, when competitiveness is lost during re-mating, i.e. $\gamma _2=0.5 \times \gamma _1$, this increases the release ratio: see Fig. 3(A)-(B), page 18. Nevertheless, the GRO-treatment is beneficial. As expected, the SIT-EPF combination performs better (compare 3(A) and 3(B)), as it does in the case of equal competitiveness. Overall, the results obtained for the critical release rate that are lower than those obtained in Dumont and Oliva (2024).

**Fig. 4:**
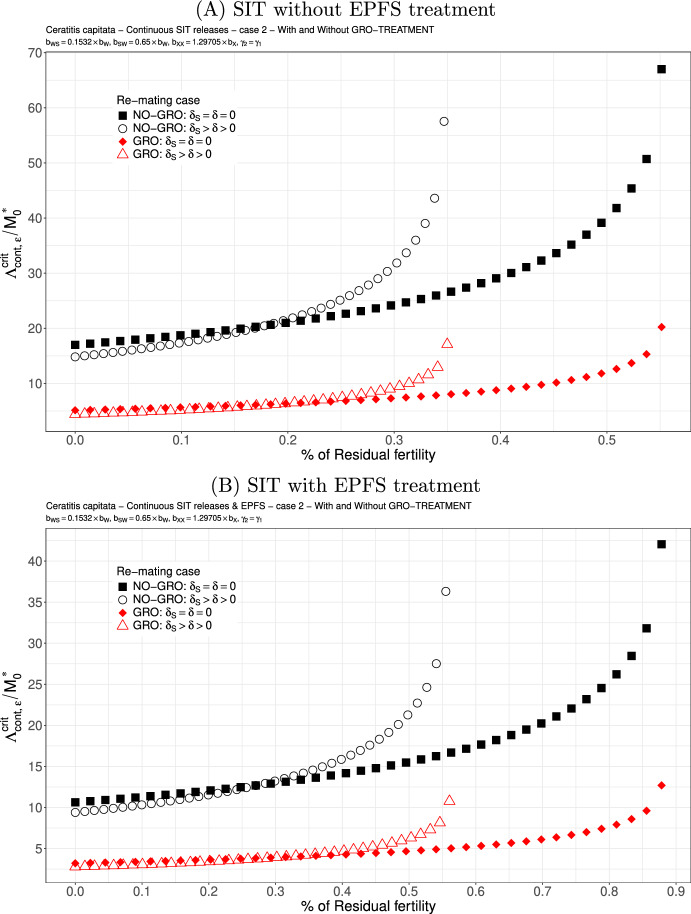
SIT Treatment against *Ceratitis capitata* with equal competitiveness, $\gamma _2=\gamma _1$ - Critical release ratio for continuous releases as a function of residual fertility - re-mating case 2 with $b_{WS}=0.1532 \times b_W$ and $b_{SW}=0.65 \times b_W$. Simulations without **(A)** and with EPFS treatment **(B)**, and different re-mating configurations: the black squares, the NO re-mating case without GRO-treatment; the black circles, the re-mating case without GRO-treatment; the red losanges, the NO re-mating case with GRO-treatment; the red triangles, the re-mating case with GRO-treatment. (Color Figure Online)

**Fig. 5:**
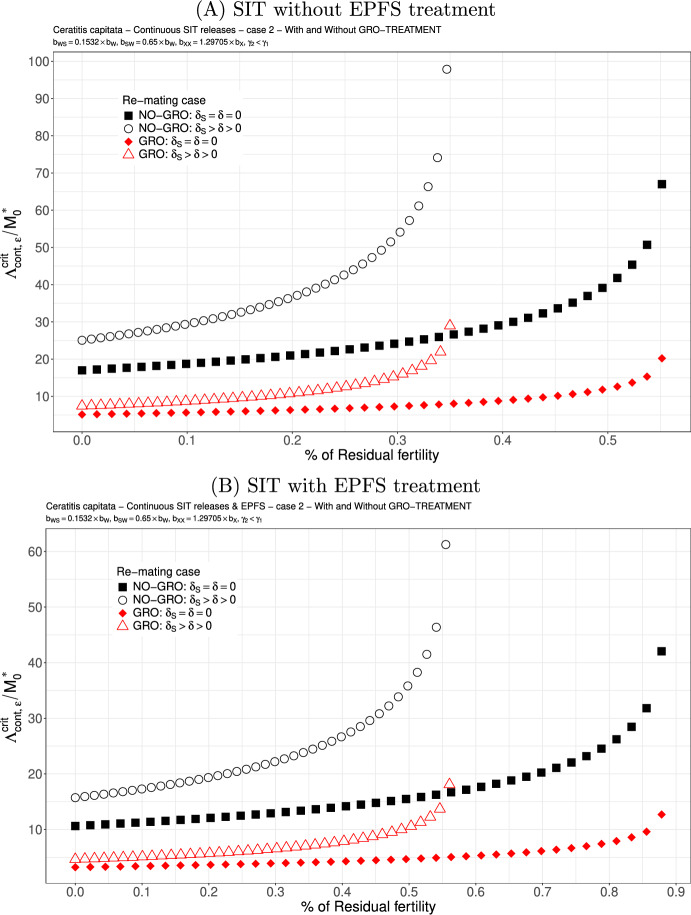
SIT Treatment against *Ceratitis capitata* with non-equal competitiveness, $\gamma _2=0.5\times \gamma _1$ - Critical release ratio for continuous releases as a function of residual fertility - re-mating case 2 with $b_{WS}=0.1532 \times b_W$ and $b_{SW}=0.65 \times b_W$. Simulations without **(A)** and with EPFS treatment **(B)**, and different re-mating configurations: the black squares, the NO re-mating case without GRO-treatment; the black circles, the re-mating case without GRO-treatment; the red losanges, the NO re-mating case with GRO-treatment; the red triangles, the re-mating case with GRO-treatment. (Color Figure Online)

Case 2. We assume that $b_{WS}=0.1532 \times b_W$ and $b_{SW}=0.65 \times b_W$. These values are taken from Lee et al. (2003): see also Dumont and Oliva (2024). Here, the second mating with a sterile male clearly decreases the rate at which viable eggs are deposited, such that we can expect this case to be favourable for re-mating. According to Figs. 4 (A)-(B), page 19, this is indeed the case as long as the residual fertility is low, less than $0.2\%$ ($0.3\%$ with EPFS). Again, the GRO-treatment is favorable and reduces drastically the critical release ratio. The SIT-EPFS combination allows to increase the residual fertility threshold like in the equal competitiveness case, and also allows to decay the release rate. However, when $\gamma _2=0.5 \times \gamma _1$, then, without or with the EPFS treatment, we loose the benefit of re-mating: see Fig. 5(A)-(B), page 20 where we can see an increase in the critical release ratio. It is also interesting to notice that Fig. 5, page 20 looks very similar to Fig. 3, page 18: it is as if the loss of competitiveness in the second mating annihilates the benefit of having $b_{WS}$ very small.

In both cases, the SIT-EPFS combination allows to release sterile males with a weaker sterility than SIT alone. This is the most interesting benefit as it allows to relax the level of radiation in order to produce sterile males with a better fitness.

Of course, once elimination is nearly reached, massive releases are no more necessary: using the strong Allee effect induced by the release of sterile males, we can switch to small releases, like in Anguelov et al. (2020), to maintain the wild population very low and to converge slowly but surely to elimination.

Note also that the critical release ratios obtained in the simulations are more or less comparable to the ratios given in Rendón et al. (2019), Table 3.

### Numerical simulations: *B. Dorsalis*

For *B. Dorsalis*, we will also consider two cases: the first one, based on the literature, where we take into account the first sperm preference, such that $b_{SW}<b_{WS}$ thanks to Zhao et al. (2013), where $b_{WS}=0.717 \times b_W$ and $b_{SW}=0.549 \times b_W$. The second case is based on recent results obtained in the AttracTIS project, in Réunion island, where, surprisingly, we found aspermia when sterile males mated with mature females: very few sterile sperms are already transferred to the mated females, such that only the fertile sperm is used to fertilize the eggs. This means that we are in a worst case, studied in Dumont and Oliva (2024), where $b_{SW}=b_{WS}=b_W$. In addition, our experiments showed that $\delta _S=\delta$.

Using the parameters values given in Table 2, page 14, we derive $\mathcal {N}_{0,W}\approx 289.27$ and $\mathcal {R}_W \approx 349.4672$ (353.8953) without (with) ME-treatment. Clearly, these basic offspring numbers are larger than those given for the medfly. This partly explained why the oriental fruit fly is so invasive, so much so that, being first detected in April 2017 in La Réunion, it has, in a few years, displaced established fruit fly populations, such as *Bactrocera zonata*, *Ceratitis quilicii*, and also *Ceratitis capitata*.

Thanks to the sterile insect parameters related to the oriental fruit fly, we derive Table 5, page 21, where the values for the sterile population basic offspring number and the critical residual sterility values, with re-mating, $\mathcal {R}_S$, without re-mating, $\mathcal {N}_{0,S}$, are computed with and without ME-treatment.

**Table 5:** SIT only - *B. Dorsalis* sterile insect’s Basic Offspring numbers and residual fertility thresholds, with and without ME-treatment

SIT	$\mathcal {N}_{S}$	$\overline{\varepsilon }$	$\mathcal {R}_{S}$	$\overline{\varepsilon }$
SM	289.2694	0.003456986	349.4672	0.002861499
SM-ME	289.2694	0.003456986	353.8953	0.002825694

Without mating, we found that $\varepsilon <1/\mathcal {N}_{0,S} \approx 0.00311129$, while, with double-mating, we have $\varepsilon <1/\mathcal {R}_S \approx 0.0025753$ (without ME-treatment) or $\varepsilon <1/\mathcal {R}_S \approx 0.002543$ (with ME-treatment). Since *B. dorsalis* has a very large basic offspring number, then the residual fertility is much more constrained. In Réunion island, in the AttracTIS project, a sterilisation at 80 Gy has been considered, such that the sterile males reached a level of sterility of $99.69\%$. That is $0.31\%$ of residual fertility. This seems to be larger than $\overline{\varepsilon }$ when re-mating is taken into account. However, our residual fertility threshold depends on sterile-mated females for which we don’t know many parameters, apart those available in the literature.

As with *Ceratitis capitata*, the SIT - EPFS combination results in clear improvements in the residual fertility threshold values. Indeed, the basic offspring number, without and with re-mating, decay substantially (see the second and fourth columns in Table 4 and compare them to Table 3), such that the constraints on the residual fertility thresholds are at least twice as large as in the case of SIT without EPFS: compare the values obtained for $\overline{\varepsilon }$ in Tables 6 and 5. Indeed, when re-mating occurs, then our sterile insects with $0.31\%$ residual fertility are admissible, because $\varepsilon =0.0031<\overline{\varepsilon }=0.0058$. Thus, from that point of view the combination SIT-EPFS is again clearly beneficial.

**Table 6:** SIT-EPFS combination - *B. dorsalis* sterile insect’s Basic Offspring numbers and residual fertility thresholds, with and without ME-treatment

SIT-EPFS	$\mathcal {N}_{S}$	$\overline{\varepsilon }$	$\mathcal {R}_{S}$	$\overline{\varepsilon }$
SM	146.7917	0.006812375	172.3567	0.005801922
SM-ME	146.7917	0.006812375	175.1342	0.005709907

We study the following two cases.

**Fig. 6:**
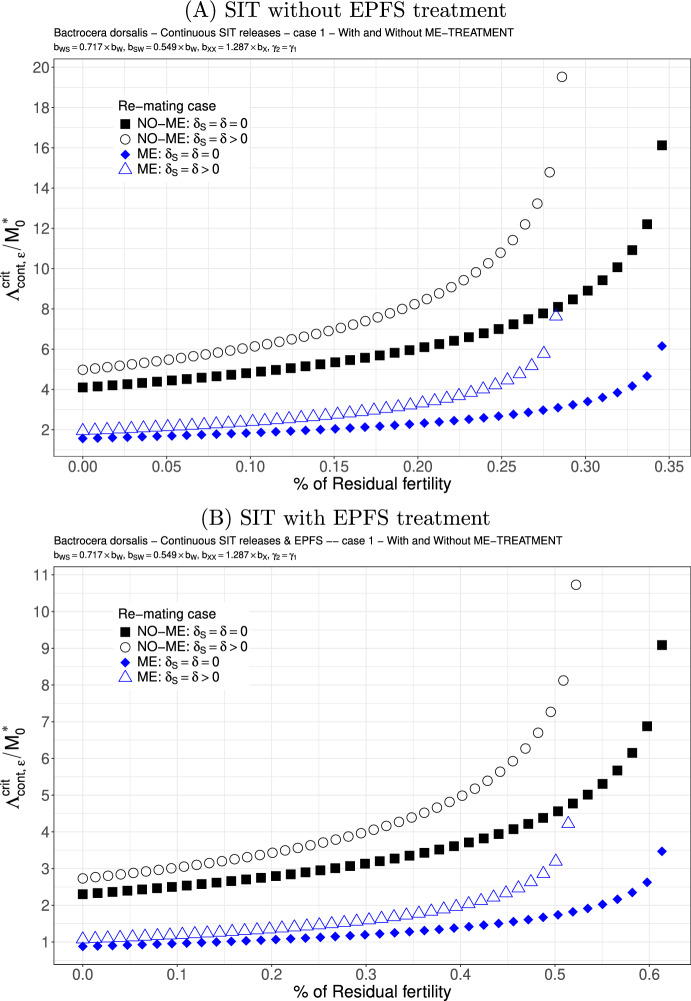
*B. dorsalis* with equal competitiveness, $\gamma _2=\gamma _1$ - Critical release ratio for continuous releases as a function of residual fertility - re-mating case 1 with $b_{WS}=0.717 \times b_W$ and $b_{SW}=0.549 \times b_W$. Simulations without **(A)** and with EPFS treatment **(B)**, and different re-mating configurations: the black squares, the NO re-mating case without ME-treatment; the black circles, the re-mating case without ME-treatment; the blue losanges, the NO re-mating case with ME-treatment; the blue triangles, the re-mating case with ME-treatment. (Color Figure Online)

**Fig. 7:**
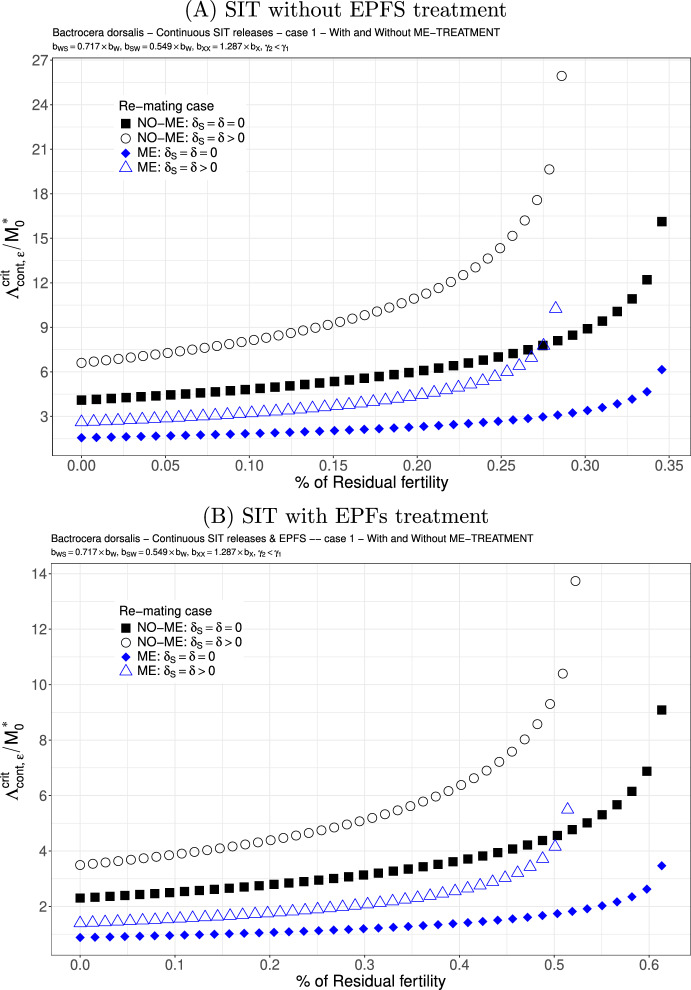
*B. dorsalis* with non-equal competitiveness, $\gamma _2=0.5\times \gamma _1$ - Critical release ratio for continuous releases as a function of residual fertility - re-mating case 1 with $b_{WS}=0.717 \times b_W$ and $b_{SW}=0.549 \times b_W$. Simulations without **(A)** and with EPFS treatment **(B)**, and different re-mating configurations: the black squares, the NO re-mating case without GRO-treatment; the black circles, the re-mating case without ME-treatment; the blue losanges, the NO re-mating case with GRO-treatment; the blue triangles, the re-mating case with ME-treatment. (Color Figure Online)

1. We consider $b_{WS}=0.717 \times b_{W}$ and $b_{SW}=0.54{9} \times b_{W}$: see Figs. 6(A)-(B) and 7(A)-(B). This is not really a good case, but this is the only published result where the fertility of double-mated *B. dorsalis*, $F_{SW}$ and $F_{WS}$, has been studied (Zhao et al. 2013). We clearly see that the first mating is very important since we have a precedence of the first sperm. This explained that in the case where sterile males are less competitive for the second mating, $\gamma _2<\gamma _1$, there is only a little increase in the release ratio threshold when re-mating occurs, at least when $\varepsilon$ is small: compare Figs. 6 and 7. Once again, the use of ME-treatment is beneficial in that it reduces the critical release rate by almost a factor 2. Finally, the SIT-EPFS combination not only improves the residual fertility threshold, $\overline{\varepsilon }$, but also decreases the critical release rate by a factor of 2: compare Figs. 6(A) and 7(A) to Figs. 6(B) and 7(B). This combination is clearly beneficial for SIT.

**Fig. 8:**
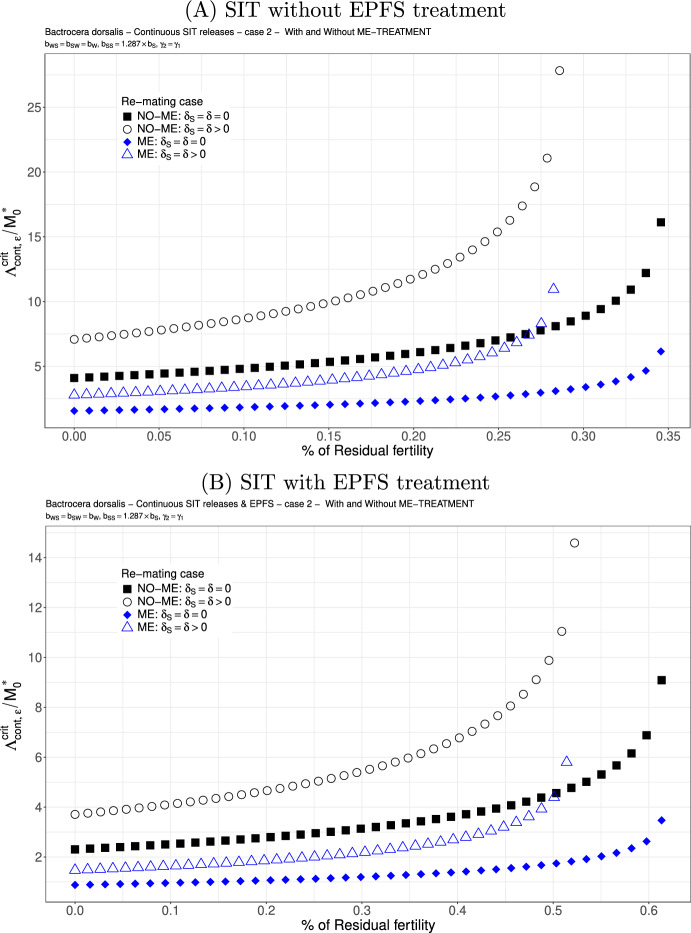
*B. dorsalis* with equal competitiveness, $\gamma _2=\gamma _1$ - Critical release ratio for continuous releases as a function of residual fertility - re-mating case 2 with $b_{W,S}=b_{S,W}=b_{W}$. Simulations without **(A)** and with EPFS treatment **(B)**, and different re-mating configurations: the black squares, the NO re-mating case without ME-treatment; the black circles, the re-mating case without ME-treatment; the blue losanges, the NO re-mating case with ME-treatment; the blue triangles, the re-mating case with ME-treatment. (Color Figure Online)

**Fig. 9:**
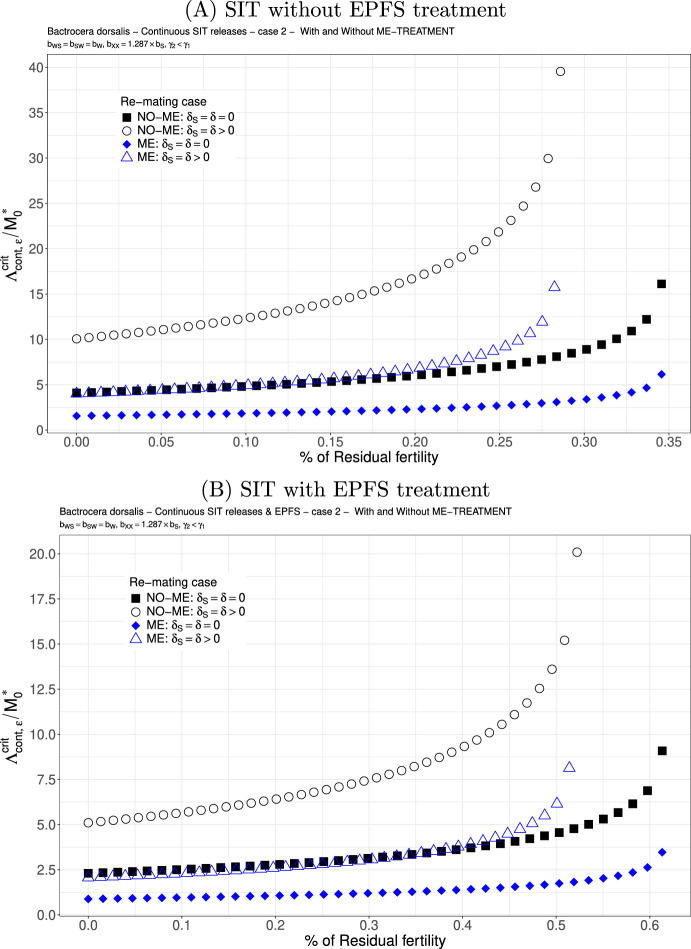
*B. dorsalis* with non-equal competitiveness, $\gamma _2=0.5\times \gamma _1$ - Critical release ratio for continuous releases as a function of residual fertility - re-mating case 2 with $b_{W,S}=b_{S,W}=b_{W}$. Simulations without **(A)** and with EPFS treatment **(B)**, and different re-mating configurations: the black squares, the NO re-mating case without GRO-treatment; the black circles, the re-mating case without ME-treatment; the blue losanges, the NO re-mating case with GRO-treatment; the blue triangles, the re-mating case with ME-treatment. (Color Figure Online)

Case 2. We now consider that $b_{WS}=b_{SW}=b_{W}$: see Figs. 8(A)-(B) and 9(A)-(B). In all figures, the ME-treatment is beneficial and reduce by a factor 2 the critical release rate. When no-remating is considered, i.e. $\delta =\delta _S=0$, then the amount of sterile males to release is underestimated compared to the case where re-mating is considered, where $\delta _S=\delta >0$. Despite the fact that this case could be considered as unfavorable for SIT because mixed-mating does not reduce the fertility, we can observe that the critical release rate ratio, $\Lambda _{\varepsilon ,cont}^{crit}/M_0^*$, is much smaller than in the Ceratitis case: this is due to the fact that the sterile males lifespan is longer. As expected, the SIT-EPFS combination improves the result, not only for the residual fertility threshold but also for the critical release rate: compare figures (A) and (B) in Figs. 8 and 9. Of course, when $\gamma _2<\gamma _1$, the critical release rate increases. However, as long as the residual fertility is less than $0.2\%$ ($0.4\%$), a critical release rate ratio equal to or greater than 7 (5), without (with) EPFS treatment, is sufficient, with or without re-mating, to drive the wild population to elimination. In addition, the EPFS treatment allows to use SIT for larger value of the residual fertility. This is really of great interest. Obviously, the release rate threshold increases with respect to $\varepsilon$. However this increase is small and almost linear for a residual fertility less than $0.25\%$ ($0.2\%$) without (with) re-mating in Fig. 8(A), while in Fig. 8(B), it is small and almost linear for a residual fertility less than $0.6\%$ ($0.5\%$) without (with) re-mating.

### Numerical simulations: periodic releases

While we have shown that the constraint on the residual fertility is similar for continuous and periodic releases, we have to solve system (13) to estimate the periodic critical release ratio $\Lambda ^{crit}_{per,\varepsilon }/M_0^*$. Like in Dumont and Oliva (2024), because the computations are long we need a fast algorithm. That is why, we will consider the nonstandard finite difference approach: see (Anguelov et al. 2012b) for an introduction and references therein. As initial condition, we consider that the wild population is at its endemic equilibrium, $\textbf{E}$, defined in (6).

We consider only case two, for both fruit flies, with equal competitiveness. For the oriental fruit fly, since the lifespan of the sterile males is longer, we can consider a 15-days releases strategy, while for the medfly we will consider a 3-days releases strategy, like in Dumont and Oliva (2024).

**Fig. 10:**
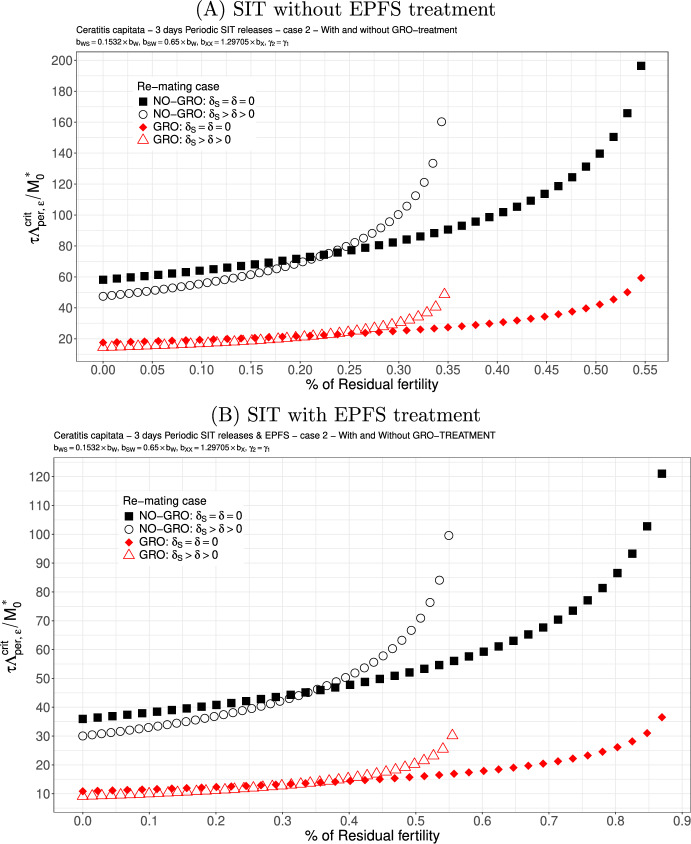
*Ceratitis capitata* with equal competitiveness, $\gamma _2=\gamma _1$ - Critical release ratio for 3-days periodic releases as a function of residual fertility - re-mating case 2 with $b_{WS}=0.1532 \times b_W$ and $b_{SW}=0.65 \times b_W$. Simulations without **(A)** and with EPFS treatment **(A)**, and different re-mating configurations: the black squares, the NO re-mating case without GRO-treatment; the black circles, the re-mating case without GRO-treatment; the red losanges, the NO re-mating case with GRO-treatment; the red triangles, the re-mating case with GRO-treatment. (Color Figure Online)

**Fig. 11:**
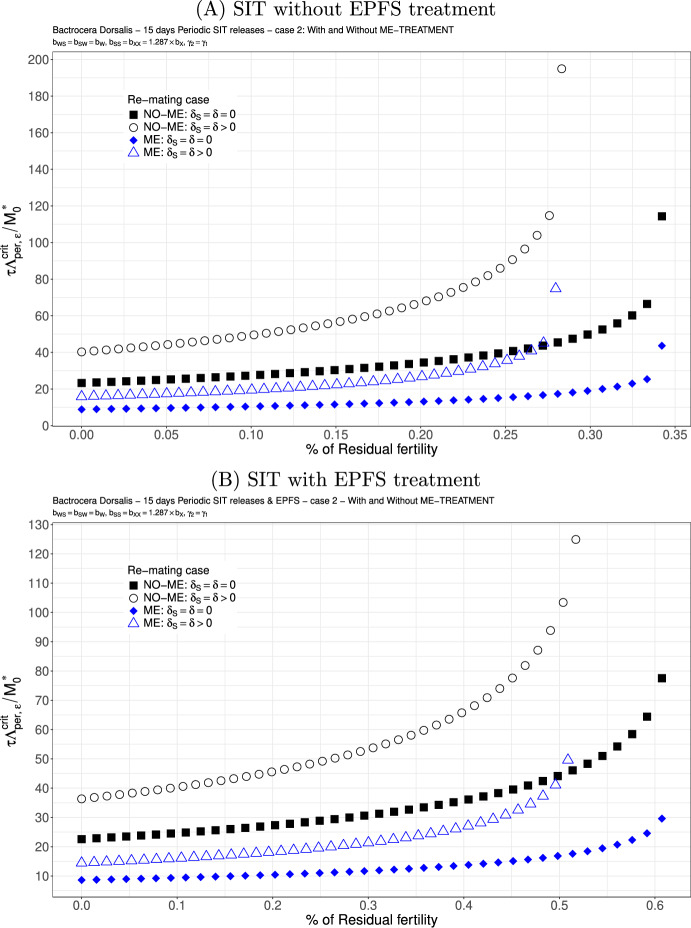
*B. dorsalis* with equal competitiveness, $\gamma _2=\gamma _1$ - Critical release ratio for 15-days periodic releases as a function of residual fertility - re-mating case 2 with $b_{WS}=b_{SW}=b_{W}$. Simulations without **(A)** and with EPFS treatment **(A)**, and different re-mating configurations: the black squares, the NO re-mating case without ME-treatment; the black circles, the re-mating case without ME-treatment; the blue losanges, the NO re-mating case with ME-treatment; the blue triangles, the re-mating case with ME-treatment. (Color Figure Online)

As can be seen in Figs. 10(A)-(B) and Fig. 11(A)-(B), we obtain similar results to that of the continuous releases, except that the ratios are larger thanks to the fact that we release every $\tau$ days. When periodic releases occur, the estimates of the critical releases rates show that in general $\Lambda _{\varepsilon ,per}^{crit}\ge \tau \Lambda _{\varepsilon ,cont}^{crit}$. As in the continuous releases case, the use of GRO-treatment (ME-treatment) clearly improves the periodic release rate threshold by a factor 3 (2). And, of course, the addition of an EPFS treatment allows to reduce again by a factor 2. Thus, in Figs. 10(A)-(B), when $\varepsilon =0$, it is clear to see that without any treatment, we need to release, at least, 50-60 times more sterile males than wild males (at equilibrium), while with a GRO-treatment, we have to release only 18 times more, and when combining with EPFS, then we have to release only 10 times more. In addition, the SIT-EPFS combination increases the residual fertility threshold by at least a factor of 2 for both fruit flies. Again, this is a major advantage for the SIT treatment, as it allows the release of sterile males that are less irradiated and therefore have better fitness, i.e., a longer lifespan, and better competitiveness.

## Conclusion

Implementing the Sterile Insect Technique (SIT) in the field is a complex process that requires monitoring various parameters in the laboratory or semi-field settings to ensure its effectiveness. While most of these parameters are part of the quality control in most SIT programs, some, particularly those related to re-mating, have not been extensively studied.

Achieving full sterility in males is a rare outcome in SIT programs. In fact, reaching complete sterility often requires unreasonably high radiation doses. Therefore, it is essential to strike a balance between acceptable residual fertility and the overall quality of the released males, which includes competitiveness and lifespan. This is well known, but in general, re-mating is never taken into account. Our study highlights that residual fertility has to be lower than expected when re-mating occurs. We derive a simple condition for the residual fertility to respect in order to guarantee that SIT is efficient: it has to be lower than a certain threshold, $1/\mathcal {R}_S$, where $\mathcal {R}_S$ is the basic offspring number related to the sterile-mated females. When residual fertility exceeds $1/\mathcal {R}_S$, SIT becomes an inefficient control strategy. As a result, the radiation dose must be carefully selected to ensure that the average residual fertility remains well below $1/\mathcal {R}_S$, without or with re-mating. Our result is far more simpler than the threshold obtained in Dumont and Oliva (2024). Thus, the larger $\mathcal {R}_S$, the lower $\varepsilon$.

Our simulations demonstrate that the critical release rate increases significantly as residual fertility rises, and this is even worst when re-mating is considered. However, when mixed-mated females, $F_{SW}$ or $F_{WS}$, have a low deposit rate, i.e. $b_{SW}<<b_W$ or $b_{WS}<<b_W$, respectively, then re-mating can be beneficial for the critical release rate: see, for instance, case 2 for the medfly.

Like in many SIT programs, we have considered additional treatments, like GRO-treatment and ME-treatment, that are known to improve substantially the efficiency of SIT by increasing the competitiveness of sterile males (Shelly et al. 2014). This is confirmed by our simulations. The competitiveness parameter has no impact on the residual fertility threshold.

Finally, we showed that the SIT-EPFS combination leads to a clear improvement of the results: the residual fertility threshold is enhanced by a factor 2 and it induces a decrease of the critical release rate. While having a lower critical release rate is useful, the main benefit is that the increase of the residual fertility threshold gives much more flexibility in the radiation doses to use, in order to produce sterile males with a good fitness.

To summarize, when re-mating occurs, our study showed that biological parameters related to sterile-mated females are central to determine the critical residual fertility and also the critical release rate. Unfortunately, we found partial or incomplete information in the literature about these parameters for the medfly and the oriental fruit fly. This is mainly due to the fact that these experiments are long, tedious, difficult, or, simply, ignored. It seems that it would be more than necessary to estimate all these parameters in order to guarantee or enhance the SIT efficacy. We considered here only single and double mating, but our model can be extended to more re-mating, requiring to obtain data related to these events, something which is not easy. In the framework of the AttracTIS project, we intend to derive estimates of most of the parameters needed to derive the residual fertility threshold, and, thus, derive the release rate threshold. In addition, the use of EPFS treatment allows to relax the constraint on the residual fertility and increase the efficacy of SIT.

Our approach could be used or adapted for other fruit flies, like *Drosophila suzukii*, for which SIT has been considered very recently (Homem et al. 2022; Chen et al. 2022), provided that all useful parameters (related to sterile-mated females) could be available.

In general, SIT models consider simplistic population dynamics with averaged parameters over the year. However, it would be interesting to consider varying parameters, or parameters depending on Temperature, humidity, etc, to understand whether variation of released male numbers over the year brings a better or similar control while reducing the program costs. Such a work was done for mosquitoes in Dumont et al. (2024).

In this study we have only considered open-loop control. However feedback control or mixed-control can be used, like in Bliman et al. (2019); Aronna and Dumont (2020), to adapt the releases ratio along the SIT control, in order to minimize the total cost of the SIT treatment.

## Figures and Tables

**Fig. 12:**
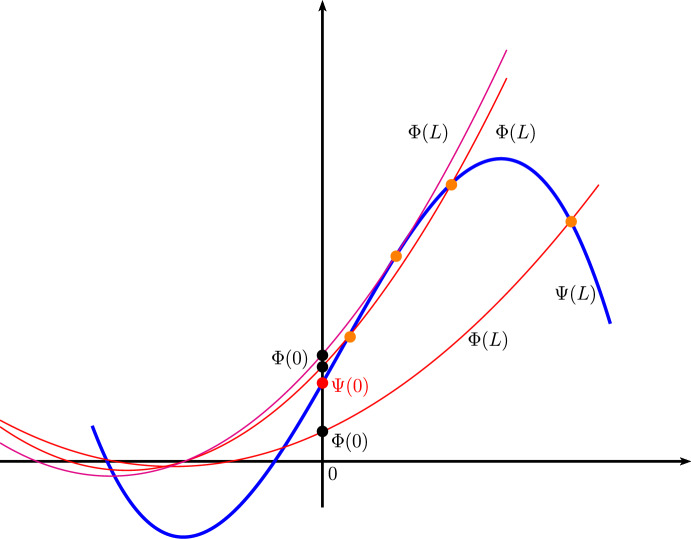
Intersections between $\Psi$ (in blue) and $\Phi$ (in red) - 3 cases with no, one or two intersections (orange bullets). (Color Figure Online)

**Fig. 13:**
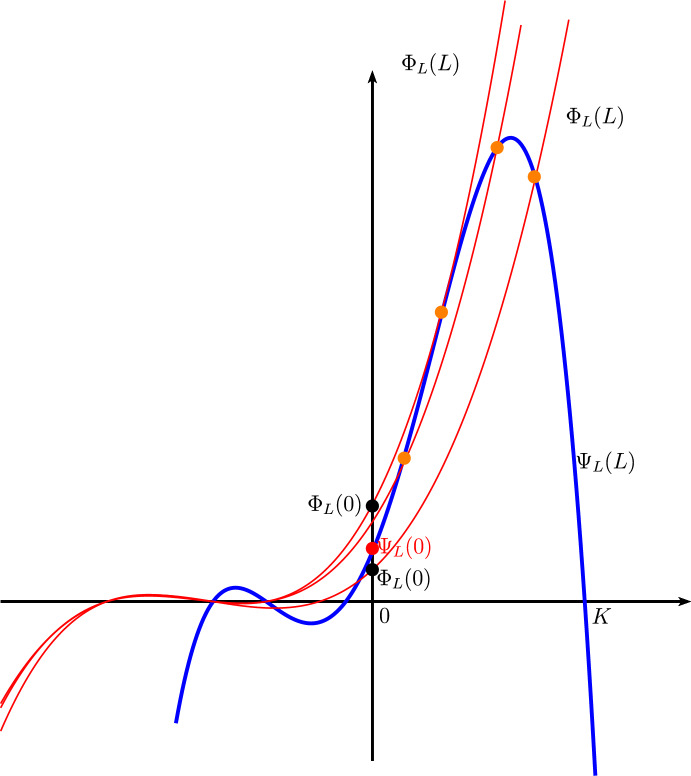
Lower system case - Intersections between $\Psi _L$ (in blue) and $\Phi _L$ (in red) - 3 cases with no, one or two intersections (orange bullets). (Color Figure Online)

## Variables and parameters

**Table 7:** Description of parameters and state variables of model (1)

Symbol	Description	Unit
*L*	Larvae stage	Individuals
*P*	Pupae stage	Individuals
$M_W$	Wild Males	Individuals
$F_W$	Once-mated females with fertilized eggs	Individuals
$F_S$	Once-mated sterile females	Individuals
$F_{SW}$	Double-mated females	Individuals
$F_{WW}$	Double-mated fertile females	Individuals
$F_{SS}$	Double-mated sterile females	Individuals
$M_S$	Sterile Males	Individuals
$\varepsilon$	Proportion of viable (hatched) eggs laid by a female $F_S$ or $F_{SS}$	-
*K*	Larvae/pupae mean carrying capacity	Individuals
$b_{W}$	Mean number of viable/hatched eggs laid by female $F_{W}$ per day	days$^{-1}$
$b_{WW}$	Mean number of viable/hatched eggs laid by female $F_{WW}$ per day	days$^{-1}$
$b_{WS}$	Mean number of viable/hatched eggs laid by female $F_{WS}$ per day	days$^{-1}$
$b_{SW}$	Mean number of viable/hatched eggs laid by female $F_{SW}$ per day	days$^{-1}$
$\varepsilon b_{S}$	Mean number of viable/hatched eggs laid by female $F_{S}$ per day	days$^{-1}$
$\varepsilon b_{SS}$	Mean number of viable/hatched eggs laid by female $F_{SS}$ per day	days$^{-1}$
$\mu _{L}$	Mortality rate of larvae, *L*	days$^{-1}$
$\nu _L$	Maturation rate from the larva stage to pupa stages	days$^{-1}$
$\mu _{P}$	Mortality rate of pupae, *P*	days$^{-1}$
$\nu _P$	Maturation rate from the pupal stage to adult stages	days$^{-1}$
*r*	Sex ratio	-
$\mu _M$	Mortality rate of wild males	day$^{-1}$
$\mu _{F}$	Mortality rate of $F_W$ females	day$^{-1}$
$\mu _{F,WW}$	Mortality rate of double-mated fertile females	day$^{-1}$
$\mu _{F,WS}$	Mortality rate of $F_{WS}$ females	day$^{-1}$
$\mu _{F,SW}$	Mortality rate of $F_{SW}$ females	day$^{-1}$
$\mu _{F,S}$	Mortality rate of $F_S$ females	day$^{-1}$
$\mu _{F,SS}$	Mortality rate of $F_{SS}$ females	day$^{-1}$
$\delta$	Re-mating rate for females $F_{W}$	day$^{-1}$
$\delta _S$	Re-mating rate for sterile females $F_{S}$	day$^{-1}$
$\mu _S$	Mortality rate of sterile male	day$^{-1}$
$\Lambda$	Sterile male release rate	individuals $\times$ days$^{-1}$
$\gamma _1$	Competitiveness parameter related to the first mating	-
$\gamma _2$	Competitiveness parameter related to the second mating	-

## Proof of Lemma 1.4

We compute the Jacobian related to system (2) at $\mathbf{{0}}_{\mathbb {R}^9}$

$$\begin{aligned} J^*= \left( \begin{array}{ccccccccc} -\left( \nu _{L}+\mu _{L}\right) & 0 & 0 & b_{W} & \varepsilon b_{S} & b_{W,W} & b_{W,S} & b_{S,W} & \varepsilon b_{S,S}\\ \nu _{L} & -\left( \nu _{P}+\mu _{P}\right) & 0 & 0 & 0 & 0 & 0 & 0 & 0\\ 0 & \left( 1-r\right) \nu _{P} & -\mu _{M} & 0 & 0 & 0 & 0 & 0 & 0\\ 0 & 0 & 0 & -\left( \delta +\mu _{F}\right) & 0 & 0 & 0 & 0 & 0\\ 0 & r\nu _{P} & 0 & 0 & -\left( \delta _{S}+\mu _{F}\right) & 0 & 0 & 0 & 0\\ 0 & 0 & 0 & \delta & 0 & -\mu _{F,WW} & 0 & 0 & 0\\ 0 & 0 & 0 & 0 & 0 & & -\mu _{F,WS} & 0 & 0\\ 0 & 0 & 0 & 0 & 0 & 0 & 0 & -\mu _{F,SW} & 0\\ 0 & 0 & 0 & 0 & \delta _{S} & 0 & 0 & 0 & -\mu _{F,SS} \end{array}\right) . \end{aligned}$$

$J^*$ is a Metzler Matrix (all off-diagonal terms are nonnegative). In order to show that $J^*$ is stable, we will decompose $J^*=M+N$, where *M* is a nonnegative matrix and *N* is Meztler-stable. If $\rho (-N^{-1}M)<1$, then $s(J^*)<0$, and thus $J^*$ is Metlzer-stable. Setting

$$ M=\left( \begin{array}{ccccccccc} 0 & 0 & 0 & 0 & 0 & 0 & 0 & 0 & 0\\ \nu _{L} & 0 & 0 & 0 & 0 & 0 & 0 & 0 & 0\\ 0 & \left( 1-r\right) \nu _{P} & 0 & 0 & 0 & 0 & 0 & 0 & 0\\ 0 & 0 & 0 & 0 & 0 & 0 & 0 & 0 & 0\\ 0 & r\nu _{P} & 0 & 0 & 0 & 0 & 0 & 0 & 0\\ 0 & 0 & 0 & \delta & 0 & 0 & 0 & 0 & 0\\ 0 & 0 & 0 & 0 & 0 & 0 & 0 & 0 & 0\\ 0 & 0 & 0 & 0 & 0 & 0 & 0 & 0 & 0\\ 0 & 0 & 0 & 0 & \delta _{S} & 0 & 0 & 0 & 0 \end{array}\right) , $$

and

$$ N=\left( \begin{array}{ccccccccc} -\left( \nu _{L}+\mu _{L}\right) & 0 & 0 & b_{W} & \varepsilon b_{S} & b_{W,W} & b_{W,S} & b_{S,W} & \varepsilon b_{S,S}\\ 0 & -\left( \nu _{P}+\mu _{P}\right) & 0 & 0 & 0 & 0 & 0 & 0 & 0\\ 0 & 0 & -\mu _{M} & 0 & 0 & 0 & 0 & 0 & 0\\ 0 & 0 & 0 & -\left( \delta +\mu _{F}\right) & 0 & 0 & 0 & 0 & 0\\ 0 & 0 & 0 & 0 & -\left( \delta _{S}+\mu _{F}\right) & 0 & 0 & 0 & 0\\ 0 & 0 & 0 & 0 & 0 & -\mu _{F,WW} & 0 & 0 & 0\\ 0 & 0 & 0 & 0 & 0 & & -\mu _{F,WS} & 0 & 0\\ 0 & 0 & 0 & 0 & 0 & 0 & 0 & -\mu _{F,SW} & 0\\ 0 & 0 & 0 & 0 & 0 & 0 & 0 & 0 & -\mu _{F,SS} \end{array}\right) , $$

where *N* is, obviously, Metzler-stable and *M* a nonnegative matrix, then, we compute

$$ -N^{-1}M=\left( \begin{array}{ccccccccc} 0 & \dfrac{\varepsilon b_{S}r\nu _{P}}{\left( \nu _{L}+\mu _{L}\right) \left( \delta _{S}+\mu _{F}\right) } & 0 & \dfrac{\delta b_{W,W}}{\left( \nu _{L}+\mu _{L}\right) \mu _{F,WW}} & \dfrac{\delta _{S}\varepsilon b_{S,S}}{\left( \nu _{L}+\mu _{L}\right) \mu _{F,SS}} & 0 & 0 & 0 & 0\\ \dfrac{\nu _{L}}{\left( \nu _{P}+\mu _{P}\right) } & 0 & 0 & 0 & 0 & 0 & 0 & 0 & 0\\ 0 & \dfrac{\left( 1-r\right) \nu _{P}}{\mu _{M}} & 0 & 0 & 0 & 0 & 0 & 0 & 0\\ 0 & 0 & 0 & 0 & 0 & 0 & 0 & 0 & 0\\ 0 & \dfrac{r\nu _{P}}{\left( \delta _{S}+\mu _{F}\right) } & 0 & 0 & 0 & 0 & 0 & 0 & 0\\ 0 & 0 & 0 & \dfrac{\delta }{\mu _{F,WW}} & 0 & 0 & 0 & 0 & 0\\ 0 & 0 & 0 & 0 & 0 & 0 & 0 & 0 & 0\\ 0 & 0 & 0 & 0 & 0 & 0 & 0 & 0 & 0\\ 0 & 0 & 0 & 0 & \dfrac{\delta _{S}}{\mu _{F,SS}} & 0 & 0 & 0 & 0 \end{array} \right) . $$

After some computations, we can show that the characteristic polynomial of $-N^{-1}M$ reduces to

$$ p(\lambda )=\lambda ^{6}\left| \begin{array}{ccc} -\lambda & 0 & \dfrac{r\nu _{P}}{\left( \delta _{S}+\mu _{F}\right) }\\ \dfrac{\delta _{S}\varepsilon b_{S,S}}{\left( \nu _{L}+\mu _{L}\right) \mu _{F,SS}} & -\lambda & \dfrac{\varepsilon b_{S}r\nu _{P}}{\left( \nu _{L}+\mu _{L}\right) \left( \delta _{S}+\mu _{F}\right) }\\ 0 & \dfrac{\nu _{L}}{\left( \nu _{P}+\mu _{P}\right) } & -\lambda \end{array}\right| =\lambda ^{6}q(\lambda ), $$

where

$$ q(\lambda )=-\lambda ^{3}+\varepsilon \mathcal {N}_{S}\lambda +\varepsilon \mathcal {N}_{S}\dfrac{\delta _{S}b_{S,S}}{b_{S}\mu _{F,SS}}. $$

Setting

$$ M_q=\left( \begin{array}{ccc} 0 & 0 & \dfrac{r\nu _{P}}{\left( \delta _{S}+\mu _{F}\right) }\\ \dfrac{\delta _{S}\varepsilon b_{S,S}}{\left( \nu _{L}+\mu _{L}\right) \mu _{F,SS}} & 0 & \dfrac{\varepsilon b_{S}r\nu _{P}}{\left( \nu _{L}+\mu _{L}\right) \left( \delta _{S}+\mu _{F}\right) }\\ 0 & \dfrac{\nu _{L}}{\left( \nu _{P}+\mu _{P}\right) } & 0 \end{array}\right) , $$

we have that $q(\lambda )$ is the characteristic polynomial of a $M_q$, a $3 \times 3$ non-negative irreducible matrix (because $(Id+M_q)^2$ is a positive matrix). From Varga (1962)[Theorem 2.7], we know that $M_q$ has a positive real eigenvalue equal to its spectral radius. Thus, we only need to focus on the positive real root, *r*, of *q*: since $q(0)<0$ and $q(1)=1-\varepsilon \mathcal {R}_S$, it is straightforward to deduce that $0<r<1$ iff $q(1)>0$, that is $\varepsilon \mathcal {R}_S<1$. Otherwise, when $\varepsilon \mathcal {R}_S>1$, then $q(1)<0$ and thus $r>1$. We deduce that when $\varepsilon$ is chosen such that $\varepsilon \mathcal {R}_S<1$, then $\rho \left( -N^{-1}M\right) <1$, that is $\mathbf{{0}}_{\mathbb {R}^9}$ is LAS for system (2). When $\varepsilon \mathcal {R}_S>1$, then $\mathbf{{0}}_{\mathbb {R}^9}$ is unstable. The result follows.

## SIT model (2) - existence of Equilibria

We are looking for a condition to have at least one positive equilibrium. Thus, we have to solve

$$ \left\{ \begin{array}{l} \left( b_{W}F_{W}+b_{WW}F_{WW}+b_{WS}F_{W,S}+b_{SW}F_{S,W}+\varepsilon b_{S}F_{S}+\varepsilon b_{S,S}F_{SS}\right) \\ \left( 1-\dfrac{L}{K}\right) -\left( \nu _{L}+\mu _{L}\right) L=0,\\ \nu _L L-\left( \nu _{P}+\mu _{P}\right) P=0, \\ \left( 1-r\right) \nu _{P}P-\mu _{M}M_W=0,\\ r\nu _{P}\dfrac{M_W}{M_W+\gamma _{1}M_{S}^*}P-\left( \delta +\mu _{F}\right) F_{W}=0,\\ r\nu _{P}\dfrac{\gamma _{1}M_{S}^*}{M_W+\gamma _{1}M_{S}^*}P-\left( \delta _{S}+\mu _{F}\right) F_{S}=0,\\ \delta \dfrac{\gamma _{2}M_{S}^*}{M_W+\gamma _{2}M_{S}^*}F_{W}-\mu _{F,WS}F_{WS}=0,\\ \delta _{S}\dfrac{M_W}{M_W+\gamma _{2}M_{S}^*}F_{S}-\mu _{F,SW}F_{SW}=0,\\ \delta \dfrac{M_W}{M_W+\gamma _{2}M_{S}^*}F_{W}-\mu _{F,WW}F_{WW}=0,\\ \delta _{S}\dfrac{\gamma M_{S}^*}{M_W+\gamma _{2}M_{S}^*}F_{S}-\mu _{F,SS}F_{SS}=0, \end{array}\right. $$

such that

$$ \left\{ \begin{array}{l} \left( b_{W}F_{W}+b_{WW}F_{WW}+b_{WS}F_{WS}+b_{SW}F_{SW}+\varepsilon b_{S}F_{S}+\varepsilon b_{S,S}F_{SS}\right) \\ \left( 1-\dfrac{L}{K}\right) =\left( \nu _{L}+\mu _{L}\right) L\\ P=\dfrac{\nu _L}{\left( \nu _{P}+\mu _{P}\right) } L, \\ M_W=\dfrac{\left( 1-r\right) \nu _{L}}{\mu _{M}}\dfrac{\nu _P}{\left( \nu _{P}+\mu _{P}\right) } L=\mathcal {N}_{M}L,\\ \dfrac{\nu _P}{\left( \nu _{P}+\mu _{P}\right) }\dfrac{r\nu _{L}}{\left( \delta +\mu _{F}\right) }\dfrac{\mathcal {N}_{M}L}{\mathcal {N}_{M}L+\gamma _{1}M_{S}^*}L=F_{W},\\ \dfrac{\nu _P}{\left( \nu _{P}+\mu _{P}\right) }\dfrac{r\nu _{L}}{\left( \delta _{S}+\mu _{F}\right) }\dfrac{\gamma _{1}M_{S}^*}{\mathcal {N}_{M}L+\gamma _{1}M_{S}^*}L=F_{S},\\ \dfrac{\delta }{\left( \delta +\mu _{F}\right) }\dfrac{\nu _P}{\left( \nu _{P}+\mu _{P}\right) }\dfrac{r\nu _{L}}{\mu _{F,WS}}\dfrac{\mathcal {N}_{M}L\gamma _{2}M_{S}^*}{\left( \mathcal {N}_{M}L+\gamma _{1}M_{S}^*\right) \left( \mathcal {N}_{M}L+\gamma _{2}M_{S}^*\right) }L=F_{WS}\\ \dfrac{\delta _{S}}{\left( \delta _{S}+\mu _{F}\right) }\dfrac{\nu _P}{\left( \nu _{P}+\mu _{P}\right) }\dfrac{r\nu _{L}}{\mu _{F,SW}}\dfrac{\gamma _{1}M_{S}^*}{\left( \mathcal {N}_{M}L+\gamma _{1}M_{S}^*\right) \left( \mathcal {N}_{M}L+\gamma _{2}M_{S}^*\right) }\mathcal {N}_{M}L^{2}=F_{SW}\\ \dfrac{\delta }{\left( \delta +\mu _{F}\right) }\dfrac{\nu _P}{\left( \nu _{P}+\mu _{P}\right) }\dfrac{r\nu _{L}}{\mu _{F,WW}}\dfrac{\left( \mathcal {N}_{M}L\right) ^{2}}{\left( \mathcal {N}_{M}L+\gamma _{1}M_{S}^*\right) \left( \mathcal {N}_{M}L+\gamma _{2}M_{S}^*\right) }L=F_{WW}\\ \dfrac{\delta _{S}}{\left( \delta _{S}+\mu _{F}\right) }\dfrac{\nu _P}{\left( \nu _{P}+\mu _{P}\right) }\dfrac{r\nu _{L}}{\mu _{F,SS}}\dfrac{\gamma _{1}\gamma _{2}\left( M_{S}^*\right) ^{2}}{\left( \mathcal {N}_{M}L+\gamma _{1}M_{S}^*\right) \left( \mathcal {N}_{M}L+\gamma _{2}M_{S}^*\right) }L=F_{SS} \end{array}\right. $$

Replacing all $F_*$ in the first equation leads to, after some straightforward computations

$$ \begin{array}{l} \left( \mathcal {N}_{W}\left( 1+\dfrac{\delta }{\mu _{F,WW}}\dfrac{b_{WW}}{b_{W}}\right) L^{2}+\left( \mathcal {N}_{W}\left( 1+\dfrac{\delta }{\mu _{F,WS}}\dfrac{b_{WS}}{b_{W}}\right) \gamma _{2}+\mathcal {N}_{S}\left( \dfrac{\delta _{S}}{\mu _{F,SW}}\dfrac{b_{SW}}{b_{S}}+\varepsilon \right) \gamma _{1}\right) \dfrac{M_{S}^*}{\mathcal {N}_{M}}L+\right. \\ \left. +\varepsilon \mathcal {N}_{S}\left( 1+\dfrac{\delta _{S}}{\mu _{F,SS}}\dfrac{b_{SS}}{b_{S}}\right) \gamma _{1}\gamma _{2}\left( \dfrac{M_{S}^*}{\mathcal {N}_{M}}\right) ^{2}\right) \left( 1-\dfrac{L}{K}\right) =\left( L+\gamma _{1}\dfrac{M_{S}^*}{\mathcal {N}_{M}}\right) \left( L+\gamma _{2}\dfrac{M_{S}^*}{\mathcal {N}_{M}}\right) , \end{array} $$

$$ \begin{array}{l} \left( \mathcal {R}_W L^{2}+\left( \mathcal {N}_{W}\left( 1+\dfrac{\delta }{\mu _{F,WS}}\dfrac{b_{WS}}{b_{W}}\right) \gamma _{2}+\mathcal {N}_{S}\left( \dfrac{\delta _{S}}{\mu _{F,SW}}\dfrac{b_{SW}}{b_{S}}+\varepsilon \right) \gamma _{1}\right) \dfrac{M_{S}^*}{\mathcal {N}_{M}}L\right. \\ \left. +\varepsilon \mathcal {R}_{S}\gamma _{1}\gamma _{2}\left( \dfrac{M_{S}^*}{\mathcal {N}_{M}}\right) ^{2}\right) \left( 1-\dfrac{L}{K}\right) =\\ =\left( L+\gamma _{1}\dfrac{M_{S}^*}{\mathcal {N}_{M}}\right) \left( L+\gamma _{2}\dfrac{M_{S}^*}{\mathcal {N}_{M}}\right) , \end{array} $$

that can be rewritten as follows

$$ Q(L)\left( 1-\dfrac{L}{K}\right) =\left( L+\gamma _{1}\dfrac{M_{S}^*}{\mathcal {N}_{M}}\right) \left( L+\gamma _{2}\dfrac{M_{S}^*}{\mathcal {N}_{M}}\right) , $$

with

$$\begin{aligned} Q(L)&=\mathcal {R}_{W}L^{2}+\left( \mathcal {N}_{w}\left( 1+\dfrac{\delta }{\mu _{F,WS}}\dfrac{b_{WS}}{b_{W}}\right) \gamma _{2}+\mathcal {N}_{S}\left( \dfrac{\delta _{S}}{\mu _{F,SW}}\dfrac{b_{SW}}{b_{S}}+\varepsilon \right) \gamma _{1}\right) \dfrac{M_{S}^*}{\mathcal {N}_{M}}L\\&\quad + +\varepsilon \mathcal {R}_{S}\gamma _{1}\gamma _{2}\left( \dfrac{M_{S}^*}{\mathcal {N}_{M}}\right) ^{2}. \end{aligned}$$

The roots of *Q* are

14 $$\begin{aligned} L_{1}^{*}=-\dfrac{\left( \mathcal {N}_{w}\left( 1+\dfrac{\delta }{\mu _{F,WS}}\dfrac{b_{WS}}{b_{W}}\right) \gamma _{2}+\mathcal {N}_{S}\left( \dfrac{\delta _{S}}{\mu _{F,SW}}\dfrac{b_{SW}}{b_{S}}+\varepsilon \right) \gamma _{1}\right) \dfrac{M_{S}^*}{\mathcal {N}_{M}}+\sqrt{\Delta }}{2\mathcal {R}_{w}},\nonumber \\ \end{aligned}$$

and

15 $$\begin{aligned} L_{2}^{*}=-\dfrac{\left( \mathcal {N}_{w}\left( 1+\dfrac{\delta }{\mu _{F,WS}}\dfrac{b_{WS}}{b_{W}}\right) \gamma _{2}+\mathcal {N}_{S}\left( \dfrac{\delta _{S}}{\mu _{F,SW}}\dfrac{b_{SW}}{b_{S}}+\varepsilon \right) \gamma _{1}\right) \dfrac{M_{S}^*}{\mathcal {N}_{M}}-\sqrt{\Delta }}{2\mathcal {R}_{W}},\nonumber \\ \end{aligned}$$

$$ \Delta =\left( \left( \mathcal {N}_{w}\left( 1+\dfrac{\delta }{\mu _{F,WS}}\dfrac{b_{WS}}{b_{W}}\right) \gamma _{2}+\mathcal {N}_{S}\left( \dfrac{\delta _{S}}{\mu _{F,SW}}\dfrac{b_{SW}}{b_{S}}+\varepsilon \right) \gamma _{1}\right) ^{2}-4\mathcal {R}_{W}\varepsilon \mathcal {R}_{S}\gamma _{1}\gamma _{2}\right) \left( \dfrac{M_{S}^*}{\mathcal {N}_{M}}\right) ^{2} $$

They are both real negative. Thus we are looking for positive roots of

16 $$\begin{aligned} \Psi (L)\equiv \mathcal {R}_{W}\left( L-L_1^*\right) \left( L-L_2^*\right) \left( 1-\dfrac{L}{K}\right) \nonumber \\ =\left( L+\gamma _{1}\dfrac{M_{S}^*}{\mathcal {N}_{M}}\right) \left( L+\gamma _{2}\dfrac{M_{S}^*}{\mathcal {N}_{M}}\right) \equiv \Phi (L).\nonumber \\ \end{aligned}$$

Thus, we can derive 3 cases as showed in Fig. 12, page 38:

1. Since $\Phi (0) < \Psi (0)$, then there always exists a positive root, $L^*$. Note also that $\Phi (0)< \Psi (0)$ is equivalent to choose $\varepsilon$ such that
  $$ \varepsilon \mathcal {R}_{S}>1 $$
  which implies that equilibrium $\textbf{0}_{\mathbb {R}^9}$ is unstable, according to Lemma 1.4, page 7.
2. Since $\Phi (0) \ge \Psi (0)$, that is $\varepsilon \mathcal {R}_{S} \le 1$, we have two cases
  - either $M_S^*$ is chosen such that we can have one intersection or two intersections between $\Psi$ and $\Phi$
  - either $M_S^*$ is chosen sufficiently large such that $\Psi$ and $\Phi$ do not intersect.
  Thus, we can deduce that there exists a critical threshold $\Lambda _{\varepsilon ,cont}^{crit}=\mu _S M_{S}^{crit}$ such that above this critical threshold no positive equilibrium can exists, only the trivial equilibrium, i.e. $\textbf{0}_{\mathbb {R}^9}$. We are not able to derive a formula for $M_{S}^{crit}$, and thus for $\Lambda _{\varepsilon ,cont}^{crit}$, but we can solve (16), page 37, to find it.

## Proof of Theorem 1.13

We follow the methodology used in Dumont and Oliva (2024), by comparing system (2) to the following monotone system, using the fact that $\dfrac{\gamma _iM_S^*}{M_W+\gamma _i M_S^*}\le 1$ for all values of $M_W\ge 0$,

17 $$\begin{aligned} \left\{ \begin{array}{l} \dfrac{dL}{dt}=\left( b_{W}F_W+b_{WW}F_{WW}+b_{SW}F_{SW}+b_{WS}F_{WS}+\varepsilon \left( b_SF_S+b_{SS}F_{SS}\right) \right) \\ \left( 1-\dfrac{L}{K}\right) -\left( \nu _{L}+\mu _{L}\right) L,\\ \dfrac{dP}{dt}= \nu _L L-\left( \nu _{P}+\mu _{P}\right) P, \\ \dfrac{dM_W}{dt}=\left( 1-r\right) \nu _{P}P-\mu _{M}M_W,\\ \dfrac{dF_{W}}{dt}=r\nu _{P}\dfrac{M_W}{M_W+\gamma _1 M_{S}^*}P-\left( \delta +\mu _{F}\right) F_{W},\\ \dfrac{dF_{S}}{dt}=r\nu _{P}P-\left( \delta _{S} +\mu _{F,S}\right) F_{S},\\ \dfrac{dF_{WW}}{dt}=\delta \dfrac{M_W}{M_W+\gamma _2 M^*_{S}}F_{W}-\mu _{F,WW}F_{WW},\\ \dfrac{dF_{WS}}{dt}=\delta F_{W}-\mu _{F,WS}F_{WS},\\ \dfrac{dF_{SW}}{dt}=\delta _{S} \dfrac{M_W}{M_W+\gamma _2 M^*_{S}}F_{S}-\mu _{F,SW}F_{SW},\\ \dfrac{dF_{SS}}{dt}=\delta _{S} F_{S}-\mu _{F,SS}F_{SS}. \end{array}\right. \end{aligned}$$

It is straightforward to check that system (2) and system (17) have the same Jacobian at $\textbf{0}_{\mathbb {R}^9}$, such that, following Lemma 1.4, page 7, we can deduce that $\textbf{0}_{\mathbb {R}^9}$ is also LAS for system (17) when $\varepsilon \mathcal {R}_{S}<1$, and unstable otherwise.

Following the same reasoning than in appendix C, we show that a positive equilibrium exists for model (17) if there exists positive roots of

$$ Q_U(L)\left( 1-\dfrac{L}{K}\right) =\left( L+\gamma _{1}\dfrac{M_{S}^*}{\mathcal {N}_{M}}\right) \left( L+\gamma _{2}\dfrac{M_{S}^*}{\mathcal {N}_{M}}\right) , $$

with

$$ Q_U(L)=\left( \mathcal {R}_{W}+\mathcal {N}_{W}\dfrac{b_{WS}}{b_{W}}\dfrac{\delta }{\mu _{F,WS}}+\mathcal {N}_{S}\dfrac{b_{SW}}{b_{S}}\dfrac{\delta _{S}}{\mu _{F,SW}}+\dfrac{\varepsilon }{\varepsilon _{\max }}\right) L^{2}+ $$

$$ +\left( \mathcal {N}_{W}\left( 1+\dfrac{b_{WS}}{b_{W}}\dfrac{\delta }{\mu _{F,WS}}\right) \gamma _{2}+\mathcal {N}_{S}\left( \dfrac{b_{SW}}{b_{S}}\dfrac{\delta _{S}}{\mu _{F,SW}}\gamma _{1}+\dfrac{\varepsilon }{\varepsilon _{\max }}\left( \gamma _{1}+\gamma _{2}\right) \right) \right) \dfrac{M_{S}^*}{\mathcal {N}_{M}}L+\varepsilon \mathcal {R}_{S}\gamma _{1}\gamma _{2}\left( \dfrac{M_{S}^*}{\mathcal {N}_{M}}\right) ^{2}. $$

It is straightforward to check that $Q_U$ admits at most two negative real roots, such that we recover a similar equation to (16), namely

18 $$\begin{aligned} \Psi _U(L)\equiv C_U\left( L-L_{1,U}^*\right) \left( L-L_{2,U}^*\right) \left( 1-\dfrac{L}{K}\right) =\left( L+\gamma _{1}\dfrac{M_{S}^*}{\mathcal {N}_{M}}\right) \left( L+\gamma _{2}\dfrac{M_{S}^*}{\mathcal {N}_{M}}\right) \equiv \Phi (L). \end{aligned}$$

where $C_U$ is the constant related to $L^2$. Like in Appendix C, we derive 3 cases, as showed in Fig. 13, page 41, where $\Psi$ is replaced by $\Psi _U$.

1. Since $\Phi _0(0) < \Psi (0)$, then (18) always admits a positive root, $L^*$. Note also that $\Phi _U(0)\le \Psi (0)$ is equivalent to choose $\varepsilon$ such that
  $$ \varepsilon \mathcal {R}_{S}>1, $$
  which implies that equilibrium $\textbf{0}_{\mathbb {R}^9}$ is unstable for system (17).
2. Since $\Phi _U(0) \ge \Psi (0)$, that is $\varepsilon \mathcal {R}_{S}\le 1$, we have two cases
  - either $M_S^*$ is small such that we can have one intersection or two intersections between $\Psi _U$ and $\Phi$
  - either $M_S^*$ is sufficiently large such that $\Psi _U$ and $\Phi$ do not intersect.
  Thus, we can deduce that there exists a critical threshold $\Lambda _{\varepsilon ,cont}^{crit}=\mu _S M_{S}^{crit}$ such that above this critical threshold no positive equilibrium can exists, only the trivial equilibrium, i.e. $\textbf{0}_{\mathbb {R}^8}$. We are not able to derive a formula for $\Lambda _{\varepsilon ,cont}^{crit}$, but we can solve (18) or, better, (16) to find the true critical threshold.

Assume $\varepsilon \in [0,\varepsilon _{\max }]$, and $\Lambda$, the release rate, greater than $\Lambda _{cont,\varepsilon }^{crit}$, such that according to the previous reasoning, only the trivial equilibrium exists, $\textbf{0}_{\mathbb {R}^9}$, that is also LAS. Thanks to the Theory of Monotone Cooperative system (Smith 2008), and following Anguelov et al. (2012a), Theorem 6 or Anguelov et al. (2020), Theorem 1, it is straightforward to deduce that $\textbf{0}_{\mathbb {R}^9}$ is not only LAS but it is GAS when $\varepsilon \le \varepsilon _{\max }$. The equilibrium $\textbf{0}_{\mathbb {R}^9}$ being GAS for the auxiliary Monotone system (17), it is also GAS for system (2), for $\Lambda >\Lambda _{cont,\varepsilon }^{crit}$.

## Proof of Lemma 2.1

We compute the Jacobian matrix for the Lower-system (L) at $\textbf{0}_{\mathbb {R}^9}$. We derive

$$ J(\textbf{0}_{\mathcal {R}^9}) = \left( \begin{array}{ccccccccc} -\left( \nu _{L}+\mu _{L}\right) & 0 & 0 & b_{W} & \varepsilon b_{S} & 0 & 0 & 0 & \varepsilon b_{S,S}\\ \nu _{L} & -\left( \nu _{P}+\mu _{P}\right) & 0 & 0 & 0 & 0 & 0 & 0 & 0\\ 0 & \left( 1-r\right) \nu _{P} & -\mu _{M} & 0 & 0 & 0 & 0 & 0 & 0\\ 0 & 0 & 0 & -\left( \delta +\mu _{F}\right) & 0 & 0 & 0 & 0 & 0\\ 0 & r\nu _{P} & 0 & 0 & -\left( \delta _{S}+\mu _{F}\right) & 0 & 0 & 0 & 0\\ 0 & 0 & 0 & \delta & 0 & -\mu _{F,WW} & 0 & 0 & 0\\ 0 & 0 & 0 & 0 & 0 & & -\mu _{F,WS} & 0 & 0\\ 0 & 0 & 0 & 0 & 0 & 0 & 0 & -\mu _{F,SW} & 0\\ 0 & 0 & 0 & 0 & \delta _{S} & 0 & 0 & 0 & -\mu _{F,SS} \end{array}\right) $$

Since $J({\textbf{0}}_{\mathbb {R}^9})$ is a Metzler Matrix, we use the same methodology used in Appendix B. We decompose $J({\textbf{0}}_{\mathbb {R}^9})$ into $M+N$ and we derive

$$ -N^{-1}M=\left( \begin{array}{ccccccccc} 0 & r\nu _{P}\varepsilon \dfrac{b_{S}}{\left( \delta _{S}+\mu _{F}\right) \left( \nu _{L}+\mu _{L}\right) } & 0 & 0 & \varepsilon \dfrac{\delta _{S}b_{S,S}}{\mu _{F,SS}\left( \nu _{L}+\mu _{L}\right) } & 0 & 0 & 0 & 0\\ \dfrac{\nu _{L}}{\left( \nu _{P}+\mu _{P}\right) } & 0 & 0 & 0 & 0 & 0 & 0 & 0 & 0\\ 0 & \dfrac{\left( 1-r\right) \nu _{P}}{\mu _{M}} & 0 & 0 & 0 & 0 & 0 & 0 & 0\\ 0 & 0 & 0 & 0 & 0 & 0 & 0 & 0 & 0\\ 0 & \dfrac{r\nu _{P}}{\left( \delta _{S}+\mu _{F}\right) } & 0 & 0 & 0 & 0 & 0 & 0 & 0\\ 0 & 0 & 0 & \dfrac{\delta }{\mu _{F,WW}} & 0 & 0 & 0 & 0 & 0\\ 0 & 0 & 0 & 0 & 0 & 0 & 0 & 0 & 0\\ 0 & 0 & 0 & 0 & 0 & 0 & 0 & 0 & 0\\ 0 & 0 & 0 & 0 & \dfrac{\delta _{S}}{\mu _{F,SS}} & 0 & 0 & 0 & 0 \end{array}\right) $$

such that straightforward calculations leads to the same characteristic polynomial, *p*, than in Appendix B. Thus, we deduce that, when $\varepsilon$ is chosen such that $\varepsilon \mathcal {R}_S<1$, then $\rho \left( -N^{-1}M\right) <1$, that is ${\textbf{0}}_{\mathbb {R}^9}$ is LAS for the Lower-system (L). When $\varepsilon \mathcal {R}_S>1$, then $\mathbf{{0}}_{\mathbb {R}^9}$ is unstable.

Following appendix D, it is straightforward to check that system (U) and system (2) have the same Jacobian matrix, at $\mathbf{{0}}_{\mathbb {R}^9}$, given in appendix B. Thus the result of Lemma (1.4) holds for system (U). Thus, we conclude that $\mathbf{{0}}_{\mathbb {R}^9}$ is LAS for systems (L) and (U) when $\varepsilon \mathcal {R}_{S}<1$. It is unstable when $\varepsilon \mathcal {R}_{S}>1$. This ends the proof.

## Proof of Proposition 2.2

Since system (U) is similar to system (17), in Appendix D, looking at a positive equilibrium is similar to the methodology developed in appendix D. Thus, we recover equality (18) where $M_S^*$ is simply replaced by ${\underline{M}}_{S}$. Thus, assuming $\varepsilon \mathcal {R}_S<1$, following the reasoning given in appendix D, and applying proposition 1.10, we deduce the existence of a critical release rate $\Lambda _{\varepsilon ,cont}^{crit}$ such that when ${\underline{M}}_{S}> \dfrac{\Lambda _{\varepsilon ,cont}^{crit}}{\mu _S}$, that is $\Lambda _{per}>\dfrac{e^{\mu _S\tau }-1}{\mu _S \tau }\Lambda _{\varepsilon ,cont}^{crit}$, then there exists only one equilibrium, $\mathbf{{0}}_{\mathbb {R}^9}$, that is not only LAS, but also GAS.
